# The Potential of Antimicrobials to Induce Thrombocytopenia in Critically Ill Patients: Data from a Randomized Controlled Trial

**DOI:** 10.1371/journal.pone.0081477

**Published:** 2013-11-28

**Authors:** Maria Egede Johansen, Jens-Ulrik Jensen, Morten Heiberg Bestle, Lars Hein, Anne Øberg Lauritsen, Hamid Tousi, Kim Michael Larsen, Jesper Løken, Thomas Mohr, Katrin Thormar, Pär I. Johansson, Alessandro Cozzi-Lepri, Jens D. Lundgren

**Affiliations:** 1 Copenhagen HIV Programme at Rigshospitalet, University of Copenhagen, Copenhagen, Denmark; 2 Department of Clinical Microbiology, Hvidovre University Hospital, Hvidovre, Denmark; 3 Department of Anesthesia and Intensive Care, North Zealand University Hospital, Hillerod, Denmark; 4 Department of Anesthesia and Intensive Care, Glostrup University Hospital, Glostrup, Denmark; 5 Department of Anesthesia and Intensive Care, Herlev University Hospital, Herlev, Denmark; 6 Department of Anesthesia and Intensive Care, Aarhus University Hospital, Aarhus, Denmark; 7 Department of Anesthesia and Intensive Care, Hvidovre University Hospital, Hvidovre, Denmark; 8 Department of Anesthesia and Intensive Care, Gentofte University Hospital, Gentofte, Denmark; 9 Department of Anesthesia and Intensive Care, Bispebjerg University Hospital, Copenhagen, Denmark; 10 Department of Transfusion Medicine and Capital Region Blood Bank, University Hospital Rigshospitalet, Copenhagen, Denmark; 11 Department of Surgery, University of Texas Medical School, Houston, Texas, United States of America; 12 Department of Virology, Royal Free and University College Medical School, London, United Kingdom; 13 Department of Infectious Diseases, University Hospital Rigshospitalet, Copenhagen, Denmark; University of Leuven, Belgium

## Abstract

**Background:**

Antimicrobial-induced thrombocytopenia is frequently described in the literature among critically ill patients. Several antimicrobials have been implicated, although experimental evidence to demonstrate causality is limited. We report, using a randomized trial, the potential of antimicrobials to induce thrombocytopenia.

**Methods:**

Randomized trial allocated patients to antimicrobial treatment according to standard- of-care (SOC group) or drug-escalation in case of procalcitonin increases (high-exposure group). Patients were followed until death or day 28. Thrombocytopenia defined as absolute (platelet count ≤100x109/L) or relative (≥20% decrease in platelet count). Analyses were performed in the two randomized groups and as a merged cohort.

**Results:**

Of the 1147 patients with platelet data available, 18% had absolute thrombocytopenia within the first 24 hours after admission to intensive care unit and additional 17% developed this complication during follow-up; 57% developed relative thrombocytopenia during follow-up. Absolute and relative thrombocytopenia day 1-4 was associated with increased mortality (HR: 1.67 [95% CI: 1.30 to 2.14]; 1.71 [95% CI: 1.30 to 2.30], P<0.0001, respectively). Patients in the high-exposure group received more antimicrobials including piperacillin/tazobactam, meropenem and ciprofloxacin compared with the SOC group, whereas cefuroxime was used more frequently in the SOC group (p<0.05). Risk of absolute and relative thrombocytopenia (RR: 0.9 [0.7-1.3], p=0.7439; 1.2 [1.0-1.4], p=0.06; respectively), as well as absolute platelet count (daily difference, high-exposure vs. SOC -1.7 [-3.8-0.5], p=0.14) was comparable between groups. In observational analyses, use of ciprofloxacin and piperacillin/tazobactam predicted risk of relative thrombocytopenia (vs. cefuroxime, RR: 2.08 [1.48-2.92]; 1.44 [1.10-1.89], respectively), however only ciprofloxacin were associated with a reduction in absolute platelet count (p=0.0005).

**Conclusion:**

High exposure to broad-spectrum antimicrobials does not result in a reduction in thrombocytopenia in critically ill patients. However, single use of ciprofloxacin, and less so piperacillin/tazobactam, may contribute to a lower platelet count.

**Trial Registration:**

ClinicalTrials.gov NCT00271752 http://clinicaltrials.gov/ct2/show/NCT00271752

## Introduction

Approximately half of the patients admitted to the Intensive Care Unit (ICU) have been reported to have thrombocytopenia [1-3] and the condition is associated with prolonged hospitalization and reduced survival rates [4]. Thrombocytopenia is often caused by severe sepsis or septic shock resulting in platelet consumption, sequestering in the spleen and microcirculation, peripheral destruction and decreased production due to hemophagocytosis [3,5,6]. In addition, several drugs administered to treat severe infection during ICU admission may cause thrombocytopenia due to bone marrow suppression or/and immune-mediated platelet destruction [7,8]. Drug-induced thrombocytopenia has been reported to be associated with a number of drugs frequently used in the ICU including heparins, analgesic and antimicrobials [3,7]. However, precisely how many episodes of thrombocytopenia is associated with the use of drugs is not well defined, but some observational studies suggest an incidence as high as 10 % [3]. 

A wide range of commonly used antimicrobials have been suspected to cause thrombocytopenia, including beta-lactams and fluorquinolones [9](10). The evidence supporting the relationship between antimicrobial agents and thrombocytopenia is mainly based on case reports and laboratory studies; it is conversely rare that platelet-reactive antibodies are directly tested for [9-11]. In the absence of a more reliable method, the gold-standard for suspecting drug-induced thrombocytopenia is the observation of an increase in platelet count after discontinuation of the drug [9,10,12]. As critically ill patients are implicitly vulnerable, the discontinuation of a potential lifesaving drug is difficult and fraught with uncertainties regarding whether fluctuations in platelet count levels thereafter is explained by the treatment or resolution of the underlying condition it was used to treat. Therefore, identification of a possible causative agent is not possible based on the available evidence, and further clarification on the contribution of antimicrobials frequently used in critically ill patients on risk of thrombocytopenia is warranted [10,13]. 

We have recently completed a large randomized controlled trial comparing outcome of two antimicrobial therapy strategies in the intensive care setting [14]. Here, we report on platelet kinetics between the two treatment groups of this trial and its relation to clinical outcome.

## Materials and Methods

### Trial design and participants

Between 2006-2009 we performed a randomized controlled trial; The Procalcitonin And Survival Study (PASS) including 1200 adult critically ill patients [14]. Patients were enrolled in the study within a maximum of 24 hours after ICU admission and randomized 1:1 to receiving either antimicrobial treatment according to standard of care (SOC group) or standard of care supplemented with daily drug escalation on the basis of procalcitonin increases (high- exposure group).

Baseline was defined as day 1 and follow-up was until death or day 28. Status along with dosage of antimicrobial therapy was registered daily and day 1 biochemistry was recorded from the samples taken at ICU admission. Primary endpoint was a comparative mortality rate between the two randomized groups (overall 28-day mortality was 31.8%). 

The primary analyses confirmed that patients randomized to the intervention group spent more days during follow-up receiving broad-spectrum antibiotics compared with the control group. Since platelet count and infection status were similar between the two groups at the time of randomization, the study design could be used to investigate the effect of exposure to large amounts of broad-spectrum antimicrobials on platelet count in critically ill patients.

In the present analysis, the effect of different antimicrobial agents on platelet count in a population of critically ill patients was explored. Four pre-selected agents were included in these analyses based on the criteria of being the most commonly used antimicrobial agents in the PASS trial and a literature review of them being implicated as possible being able induce thrombocytopenia. Thrombocytopenia defined as absolute (one platelet count ≤ 100 x 10^9^/L) or relative (≥20 % decrease in platelet count from study entry) was investigated. The study was approved by the scientific ethical committee and The Danish Data Protection Agency, and complied with the Second Declaration of Helsinki. Written informed consent was obtained from all patients. The primary trial protocol and analysis plan are available in the online supplement. Analysis of high exposure vs. SOC was by intention to treat: NCT00271752.

#### Statistical analysis

Risk of thrombocytopenia was analyzed by a Kaplan-Meier/hazard function and corresponding Wilcoxon tests. Wilcoxon test was used instead of the more commonly used log-rank test because it was felt to be important to use a test giving more weight to early events. In patients with platelets >100 10^9^/L at study entry time from study entry to a decrease to absolute thrombocytopenia and time to a decrease to absolute thrombocytopenia or death was investigated. Similarly, analyses were performed for the time from study entry to relative thrombocytopenia and time to relative thrombocytopenia or death. Finally, we investigated whether thrombocytopenia status within day 1-4 affected risk of death from day 5-.28 among those still alive at day 4. For analyses not involving death as an endpoint, follow-up time accrued from study entry to event or last available platelet count, whichever occurred first. For analyses involving death, follow-up time between the date of last available platelet counts and the time a person was last known to be alive was added.

Multivariable analyses were conducted on the risk of thrombocytopenia after enrollment in the study using standard survival analysis method such as Cox regression and Poisson regression models. The association between current drug use and the risk of developing thrombocytopenia was investigated using a Poisson multivariable regression analysis. We compared single antimicrobial use in separate models with the use of cefuroxime as the comparator group. Thus, for example, when evaluating the difference between ciprofloxacin and cefuroxime, we compared ciprofloxacin use (as single drug or in combination with others but cefuroxime) and cefuroxime use (as single drug or in combination with others but cefuroxime). Dichotomized covariates were included in the model using the median value.

A mixed quadratic effect model was employed to assess the response in platelet count in the raw scale using in separate models, values measured during follow-up. The quadratic model seemed to fit the data better than a simple mixed linear model. In univariable analysis, a model including the exposure of interest, a linear term and a quadratic term for time as well as an interaction term between time and the exposure of interest was included. Thus for example, if the exposure of interest was the presence of septic-shock at entry, the linear term of the model would estimate the daily change in platelets comparing people with or without septic shock at entry. A separate model was fitted for each of the exposure factors of interests. The mixed model was a random effect model with both random intercept and slope.

In both the mixed model and the survival analyses we attempted to control for potential confounding factors. Covariates included in the models were; randomization group (high-exposure group vs. SOC group), age ≥65 vs. <65 years, gender, APACHE II score (≥20 vs. <20), severe sepsis/septic shock at randomization (yes or no), surgical vs. medical patient, BMI (≥30 vs. <30) and chronic disease (Charlson score (≥2 vs. <2). No adjustment was made for current use of specific antimicrobials (exposure of interest). Adjusted analysis was performed for all covariates aside from the randomized groups. In order to assess the consistency of the observed differences between specific antimicrobials, we performed separate analyses in the subsets of people with or without septic shock at entry. Also, we formally tested for evidence of interaction by including an interaction term in the models. All statistical analyses SAS Enterprise 4.2. All reported p values are two sided using a level of significance of 0.05.

Sample size calculations were performed; Chi-square test for the randomized groups with a significant level at 5% and a power of 80%. Using a premise of the endpoint occurring in 20% of patients in the SOC group and 1147 patients were included in the analysis, a detection limit (two-sided) for relative risk of 1.5 in the high-exposure group was established.

## Results

### Study population

1147 of the 1200 randomized patients were included for these analyses. 53 patients were excluded due to missing data on platelet counts at study entry (i.e. within 24 hours of ICU admission) (n=49) and “surgical vs. “medical” status (n= 4) (Supp. material-flowchart Figure S1). Patients’ characteristics at study entry were comparable between the groups, thus the balance achieved by randomization was not severely affected by the exclusions (Table **1**). 

**Table 1:** Main characteristic at study entry.

		**Randomization group**			
		**SOC (n=571)**	**High-exposure (n=576)**	**Total (n=1147)**	p-value*
**Gender**	Male, n (%)	317 (50.0)	318 (50.0)	635 (55.4)	0.9165
**Age, years**	Median (IQR)	67 (59-75)	67 (58-76)	67 (58-75)	0.4337
	>65	320 (56.0)	325 (56.4)	645 (54.9)	0.8965
**Body Mass Index, kg/m^2^**	Median (IQR)	24.7 (22.2-27.8)	24.8 (22.5-28.1)	24.7 (22.2 - 27.8)	0.3683
	>30, no. (%)	96 (16.8)	104 (18.1)	200 (17.4)	0.5795
**Severe sepsis/septic shock**	no. (%)	196 (34.3)	225 (39.1)	421 (36.7)	0.2408
**APACHE II score**	Median (IQR)	18 (13-24)	18 (13-24)	18 (13-24)	0.5218
	≥20, no. (%)	232 (40.6)	215 (37.3)	447 (39.0)	0.2518
**Surgical patient**	no. (%)	166 (29.1)	158 (27.4)	324 (28.2)	0.5374
**Platelet count (x10^9^/L)**	Median (IQR)	204 (132-301)	202 (117-295)	203 (126-298)	0.2408
	≤100, no. (%)	93 (16.1)	118 (20.0)	211 (18.4)	0.5692
**Charlson score**	Median (IQR)	1 (0-2)	1 (0-2)	1 (0-2)	0.2631
	>1, no. (%)	207 (36.3)	193 (33.5)	400 (34.9)	0.3298

IQR, interquartile range.

Time of study entry (i.e. within 24 hours of ICU admission) Severe sepsis/septic shock defined according to the American College of Chest Physicians/Society of Critical Care Medicine.

### Use of antimicrobial agents

**Figure 1:**
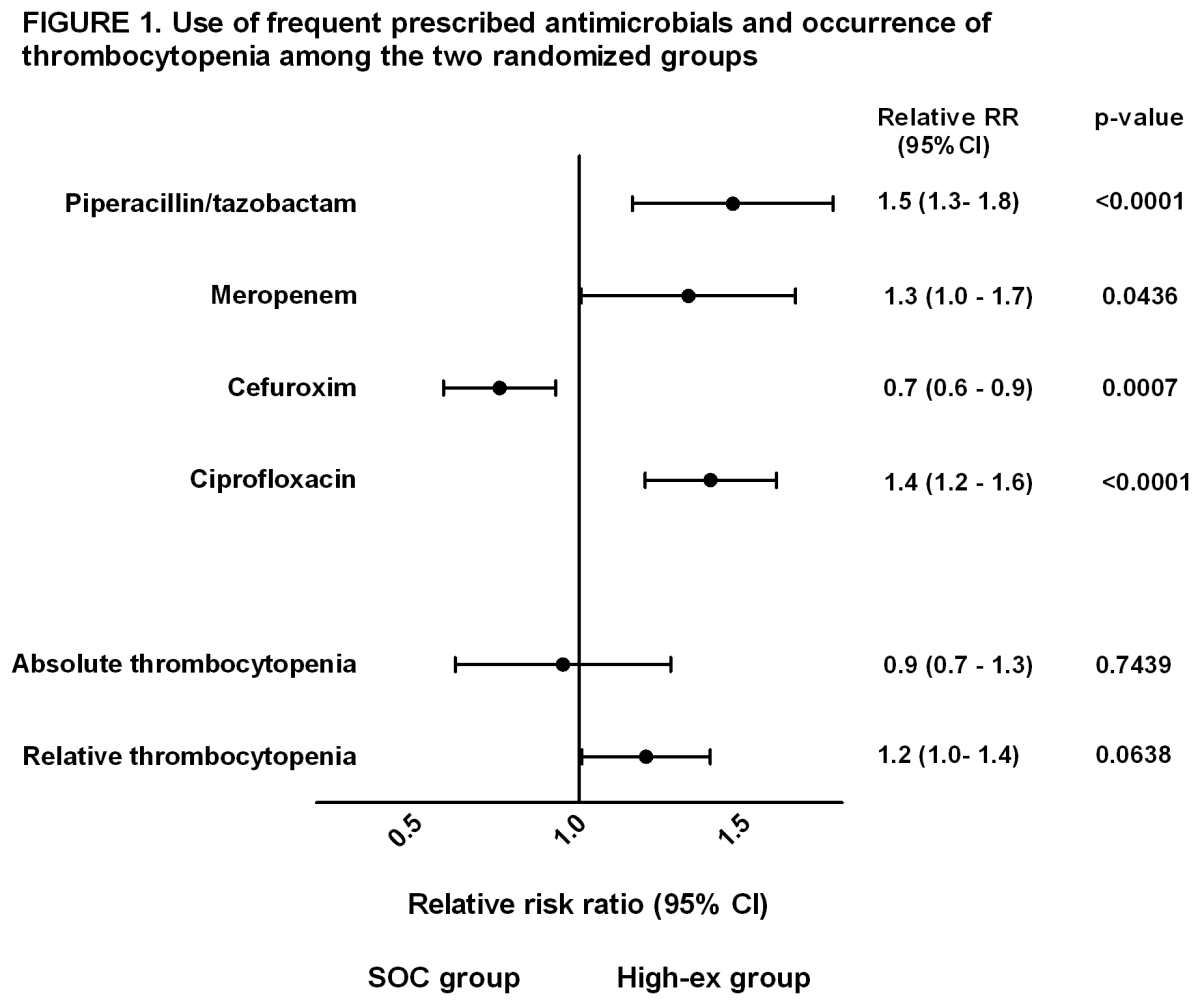
Use of frequent prescribed antimicrobials in the PASS study and occurrence of absolute and relative thrombocytopenia among the two randomized groups. Unadjusted analysis displaying the use of antimicrobials and occurrence of thrombocytopenia among the two randomized groups displayed as relative rate ratio (RR) during 28 day follow-up. Absolute (one platelet count < 100 x 109/L) or relative (>=20 % decrease in platelet count from ICU admission) thrombocytopenia. RR-Ratio >1.0 indicates that the high-ex. group have a relatively higher risk of occurrence of the asses variable and RR-Ratio <1.0 indicates relatively higher risk of patients in the SOC group of occurrence of the asses variable. RR-ratio=0 indicates no difference between the two groups.

During follow-up patients spent 11556 (44.8%) of 25776 days receiving antimicrobials. Patients in the high-exposure group received substantial more antimicrobials compared to the SOC group (6024 (23%) vs 5532 (21%) days, *p<0.0001*). Piperacillin/tazobactam, meropenem, cefuroxime and ciprofloxacin were the mostly used agents with; 3759 (15%), 1981 (8%), 2986 (12%) and 5660 (22%) days of total follow-up, respectively. Piperacillin/tazobactam, meropenem and ciprofloxacin were used more frequently among patients in the high-exposure groups compared to the SOC group (Figure **1**). Conversely, in the SOC group, cefuroxime was preferentially used (Figure **1**). 

### Thrombocytopenia during ICU hospitalization

During follow-up, 337 patients (35.2% of patients at risk [95% CI: 33.2 to 37.2]) suffered absolute thrombocytopenia (one platelet count ≤ 100 x 10^9^/L) of which 211 (18.0 % of patients at risk [95% CI: 16.2 to 19.0]) patients already had this condition at study entry. In 298 of patients with absolute thrombocytopenia (27.8 % of patients at risk [95% CI: 26.4 to 29.2]), the episode occurred within the first 4 days after study entry. 

A total of 435 (57.0 % [95% CI: 55.3 to 58.9]) suffered relative thrombocytopenia (≥20 % decrease in platelet count from study entry) during follow-up. In 351 of patients with relative thrombocytopenia (35.4% of patients at risk [95% CI: 33.9 to 36.9]), the decrease happened within the first 4 days. Of these, 98 (27.9%) never reached a platelet count ≤ 100 x 10^9^/L.

### Thrombocytopenia associated with antimicrobial strategies in the two original randomized groups

The median platelet counts did not differ significantly between the randomized groups at any time day 1-28 (*p ≥ 0.08*). Also, no difference in the occurrence of absolute and relative thrombocytopenia was observed between patients in the SOC group and in the high-ex group during 28 day follow-up (Figure **1** **.**).

In order to identify a potential effect on platelet count not necessarily resulting in thrombocytopenia, an adjusted mixed effect models was built including platelet count during the first week as well as entire follow-up. Comparing SOC vs. high-exposure group daily decrease in platelet count (x 10^9^/L) did not differ (day 1-7; -1.1 [95%CI:-2.5 to 4.6], *p=0.5613* and day 1-28; -1.7 [95%CI:-3.8 to 0.5], *p=0.1403*, respectively).

### Thrombocytopenia associated with single antimicrobial agents

Whether use of the four common used antimicrobials was associated with the platelet count was investigated in a multivariable model using the entire cohort in an observational design. No association was identified between absolute thrombocytopenia and any of the drugs in question (Table **2**). However, current use of ciprofloxacin and piperacillin/tazobactam were both identified as predictors of relative thrombocytopenia (using cefuroxime as the comparator, rate ratios (RR): 2.08 [95% CI: 1.48 to 2.92] *p<0.0001*; 1.44 [95% CI: 1.10 to 1.89], *p=0.0122*) (Table **2**). 

**Table 2:** Rate Ratio of absolute and relative thrombocytopenia.

	**Absolute thrombocytopenia (95% CI)**		**Relative thrombocytopenia (95% CI)**	
**Antimicrobials**	Unadjusted	Adjusted*	Unadjusted	Adjusted*
**None**	0.31 (0.17 to 0.54)	0.26 (0.15 to 0.48)	0.39 (0.28 to 0.53)	0.38 (0.28 to 0.53)
**Piperacillin/Tazobactam**	0.93 (0.58 to 1.50)	0.86 (0.51 to 1.44)	1.61 (1.24 to 2.10)	1.44 (1.10 to 1.89)
**Meropenem**	1.10 (0.63 to 1.92)	1.09 (0.59 to 2.05)	1.46 (1.07 to 1.99)	1.36(0.96 to 1.92)
**Ciprofloxacin**	1.62 (0.93 to 2.82)	1.59 (0.88 to 2.90)	2.45 (1.76 to 3.41)	2.08 (1.48 to 2.92)
**Cefuroxime**	1.00	1.00	1.00	1.00

A separate Poisson model for each antibiotic (used alone or in combinations non including cefuroxime) compared to people receiving cefuroxime (used alone or in combinations non including the antibiotic in question)

* Adjusted for randomization group, site of randomization, age and gender, BMI, type of patient (surgical vs. medical), APACHE score, Charlson score, septic shock and baseline platelet count

In order to account for competing risk of 28-day mortality, a composite endpoint of “thrombocytopenia (relative or absolute) or death” was created. After adjusting for confounders, ciprofloxacin and piperacillin/tazobactam increased the risk of absolute thrombocytopenia (RR: 1.73 [95% CI: 1.28 to 2.33] *p<0.0001*; 1.30 [95% CI: 1.02 to 1.67]) when combined with death. Patients currently receiving ciprofloxacin were at increased risk of reaching the composite endpoint of “relative thrombocytopenia or death (RR: 1.85 [95% CI: 1.06 to 3.24]).

Since combination of ciprofloxacin and piperacillin/tazobactam is frequent, we created a separate model to compare the risk of relative thrombocytopenia associated with currently receiving piperacillin/tazobactam with or without ciprofloxacin; no difference was found between use of the single drug or as part of combination not including ciprofloxacin. Receiving piperacillin/tazobactam in combination with ciprofloxacin increased the risk; piperacillin/tazobactam vs. piperacillin/tazobactam + ciprofloxacin (RR: 1.15 [95% CI: 0.60 to 2.21] vs. 2.17 [95% CI: 1.10 to 4.26].

**Figure 2:**
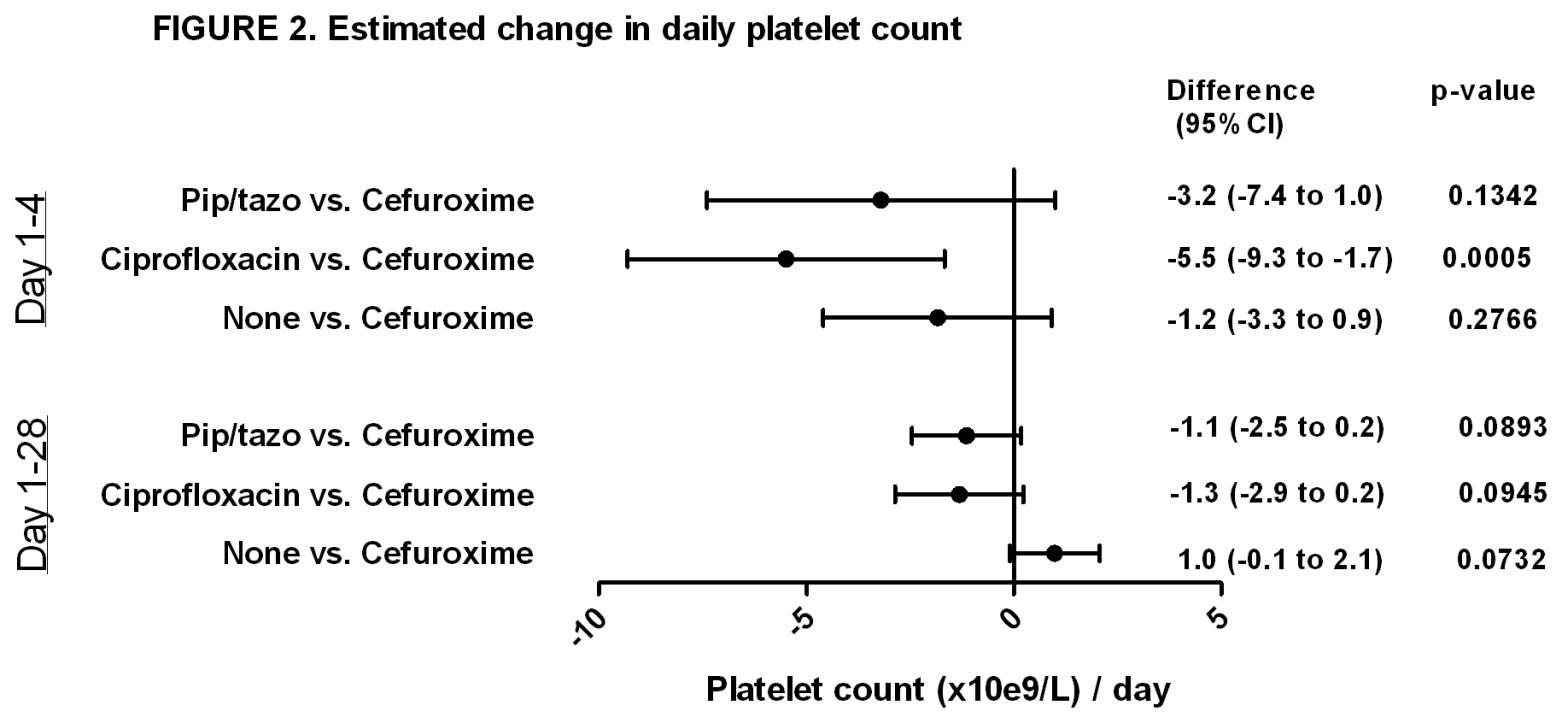
Estimated change in daily platelet count. Mixed model adjusted for the following time fixed variables: randomisation group, age, gender, BMI, severe sepsis/septic shock at ICU admission, APACHE II score, surgical vs. medical patients. Time-updated use of antimicrobials was included in the model. Ciprofloxacin, Piperacillin/tazobactam (pip/tazo) (used alone or in combinations not including cefuroxime) and none (no antimicrobials) compared to people receiving cefuroxime (used alone or in combinations non including the antibiotic in question).

To investigate potential drug effect on the absolute platelet count we included the four aforementioned antimicrobials in a mixed effect model. No significant difference was detected between cefuroxime and no antimicrobials and only the single use of ciprofloxacin day 1-4 was associated with a decline in platelet count compared to cefuroxime (Figure **2**).

A stratified analysis was performed in the subsets of with or without severe sepsis/septic shock at study entry. No interaction was detected for any of the drugs in question (piperacillin/tazobactam *p= 0.1844*; meropenem *p=0.3806*; ciprofloxacin *p=0.0747*; cefuroxime *p=0.2020*).

### Severe infection associated with absolute and relative thrombocytopenia

At study entry 421 patients was diagnosed with severe sepsis/septic shock. Of them, 170 (49.6% of patients at risk [95% CI: 47.0 to 52.2]) and 143 (41.7% of patients at risk [95% CI: 38.8 to 41.7]) suffered absolute and relative thrombocytopenia, respectively, within the first 4 days after study entry. Among patients *without* severe infection 160 (26.6% of patient at risk [95% CI: 24.8 to 28.4]) and 86 patients (14.3% of patient at risk [95% CI: 12.5 to 16.1]) were diagnosed with absolute or relative thrombocytopenia, respectively, within the first 4 days.

Patients with severe sepsis/septic shock had lower median platelet counts (x 10^9^/L) within the first four days after study entry compared with patients without this condition; 157 [95% CI: 77 to 267] vs. 221 [95% CI: 149 to 326] respectively, *p<0.0001*. 

Severe sepsis/septic shock predicted absolute and relative thrombocytopenia (RR: 1.7 [95% CI: 1.1-2.6], *p=0.0144*; 1.8 [95% CI: 1.4-2.1], *p<0.0001*, respectively) and the condition resulted in a larger daily change in platelet count (x 10^9^/L) the first 4 days after study entry compared to not suffering this condition (difference: -4.8 [95% CI:-8.7 to -1.0] *P=0.0129*). These findings were maintained taking exposure to antimicrobials into account.

### Prognostic value of absolute and relative thrombocytopenia

Thrombocytopenia (absolute and relative) occurred during the first 4 days after study entry in the majority of patients. By a multivariable analysis, both absolute and relative thrombocytopenia day 1-4 after study entry predicted 28 days mortality (HR: 1.67 [95 % CI: 1.30 to 2.14] *p<0.0001*; 1.71 [95% CI: 1.30 to 2.30] *P<0.0001*, respectively). 

## Discussion

By using data from a randomized controlled trial that per design lead to an experimental separation of exposure to antimicrobials proposed to cause thrombocytopenia, we were not able to detect a significant difference in risk of thrombocytopenia between those highly and not highly exposed to antimicrobials. Importantly, this comparison was well-powered to detect even small differences in platelet counts. In subsequent observational analysis after combining the entire cohort, ciprofloxacin, and less so piperacillin/tazobactam, was found to be associated with thrombocytopenia compared with either using cefuroxime or no antibiotics. However, the size of the effect was modest. Therefore, our findings suggests that antimicrobial drugs only marginally affect platelet counts in critically ill patients, and other factors such as severe infection, older age, surgery and chronic disease should be suspected as more likely candidates explaining this condition. 

Prior knowledge on the ability of antimicrobials to induce thrombocytopenia is based on case studies [7,9,10] subject to reporting bias and inability of confirming causality. As such, our findings from the analysis of the randomized comparison contribute with novel information to quantify the risk associated with use of antimicrobials suspected to cause thrombocytopenia. The observation period in this study was 28 days but the putative antimicrobials were only used for a fraction of this time. This means that the endpoint of absolute and/or relative thrombocytopenia would provide a signal of excess risk if an agent actually resulted in the endpoint. Relative thrombocytopenia was observed more frequently than absolute thrombocytopenia, and is a more sensitive marker of more subtle changes in platelet count. Although not statistically significant, using this outcome there was a trend for an excess risk (p=0.06) in the high-exposure group consistent with the a priori hypothesis that thrombocytopenia would develop more frequently in the arm of the study. 

The second part of the analysis combining both arms of the cohort was observational in nature. As observational analysis is subject to the same biases as those presented in prior reports, findings from these analyses should be interpreted carefully. The purpose of these analyses was to assess whether our data supported the postulated associations between specific antimicrobials and thrombocytopenia. Consistent with the literature [10,15,16], current use of ciprofloxacin and/or piperacillin/tazobactam was associated with relative thrombocytopenia, whereas none of the drugs were associated with absolute thrombocytopenia. When investigating the association between current use of antimicrobials and risk of thrombocytopenia or death confounding by indication is likely. Certain agents might have been used more in patients with worse prognosis resulting in higher likelihood of thrombocytopenia or death. Although estimates from the Poisson regression analysis were adjusted for current prognostic factors, it is conceivable that other factors associated with disease severity (e.g. specific type of infection) and use of specific antimicrobials was undetected. This is most likely the explanation why piperacillin/tazobactam and ciprofloxacin were found to be associated with absolute thrombocytopenia only when using the composite endpoint. 

Since ciprofloxacin is often prescribed in combination with piperacillin/tazobactam we hypothesized that the association between piperacillin/tazobactam and relative thrombocytopenia was driven by the thrombocytopenic induction of ciprofloxacin. Therefore, we built a model including drugs in combination with or without ciprofloxacin. The results indicated that such an effect was present underlining that the use of ciprofloxacin in any combination affects platelet count. 

Median APACHE II score did not differ among the patients receiving the single antimicrobial agents. This implies that the observed association between ciprofloxacin and relative thrombocytopenia cannot be attributed to patients receiving ciprofloxacin having a worse prognosis compared to patients receiving other types of antimicrobials.

In other analyses assessing average change in platelet counts a more mixed picture emerged. In analysis using all platelet measurements over the entire study period of 28 days, no difference in average platelet count was detected. However, an association was detected between use of ciprofloxacin and lower average platelet count when including only platelet measurements during the first four days after study entry just after patients were admitted to the ICU. This signal was consistent with observed excess risk of relative thrombocytopenia associated with ongoing use of this drug. We projected a priori that if such a signal would be found, it was more plausible that it would be detected in the analyses using only measurements in the first four days, as the antimicrobials was more frequently used early on after ICU admission compared to later on during follow-up. 

In a systematic review including 515 patient with drug-induced thrombocytopenia, time from exposure to initial occurrence of thrombocytopenia was between 1 day and up to 3 years [17]. As the knowledge regarding timing of ciprofloxacin- and piperacillin/tazobactam-induced thrombocytopenia is based on case reports, it is not possible to reliable estimate the time course of thrombocytopenia from that review. However, interestingly, several other published reports regarding antimicrobial-induced thrombocytopenia describes decreasing platelet count and development of thrombocytopenia within 4 days after exposure to ciprofloxacin [15,18-20] and piperacillin/tazobactam [21-23] consistent with our findings. The great diversity in timing is most likely explained by varying mechanisms causing drug-induced thrombocytopenia [7,10],including that prior exposure may have already sensitised some patients leading to more rapid effects upon reexposure [13]. Our study design did not allow us to address the potential mechanisms that may underlie our observation suggesting that exposure to specific antimicrobials may lead to smaller reductions in platelet counts. 

Of note, in our study we did not find any indication that meropenem or cefuroxime adversely affected platelet count. The evidence in the literature supporting the association between these two agents and thrombocytopenia is limited with only a small number of exceptions based on case reports and small case series [7,10,24]. 

There could be several reasons why ciprofloxacin in particular seems to affect the platelets in critically ill patients. Critically ill patients might have a higher cumulative dose of ciprofloxacin due to impaired kidney function [25] and high age resulting in reduced glomerulo filtration rate [26]. A potentially compounding factor may be severe infection known to induce platelet dysfunction which a great number of the patients in our study suffered from [6]. The precise mechanism whereby ciprofloxacin might affect the platelets is unknown. However, a structural link between fluoroquinolones and quinines (quinine differs in the extra side chain at position 4 where quinolones have an oxygen molecule) has been purposed to explain the ability of the drug to lower the platelet count [15,27,28]. Thus, fluoroquinolones represent the classical example of drug dependent immune-mediated thrombocytopenia possibly explaining why this particular agent displayed an association with platelet count decrease in our study.

Thrombocytopenia is frequently observed in critically ill patients and unsurprisingly approximately 1/3 of all patients in our study were diagnosed with absolute thrombocytopenia. Consisting with prior findings, most episodes occurred within the first 4 days after ICU admission [29]. Patients with absolute thrombocytopenia within the first few days after ICU admission had a 67% relative elevated risk of dying within the 28 day study period. It is possible that the platelet count is a surrogate for immune activation or severe infection. On the other hand it could be, that having a large number of well-functioning platelets may assist in improving survival from critical illness. Absolute platelet counts ≤100 x 10^9^/L increases the risk of bleeding why prevention is important [1]. Also, bacterial invasion triggers platelet activation and secretion of antimicrobial peptides [30]. A decrease in platelet count might protract clearance of infection [31]. Hence, the cell is not only pivotal as an actor in hemostasis but may also modify the immune response. 

We also explored the prognostic effect of relative thrombocytopenia. A cut-off of 20% was chosen in order to include as many patients as possible for these analyses and at the same time avoid that a decrease in platelet count was due to measurement variability. We found that that a 20% decrease in platelet count was associated with 28-day mortality; Three-quarters of patients with relative thrombocytopenia never reached a platelet count ≤ 100 x 10^9^/L but still retained a 71% increased risk of death if the episode occurred within day 1-4 after study entry. Therefore, this finding suggest that platelet count decrease influence prognosis in critically ill patients even when the threshold for absolute thrombocytopenia is never reached [32]. 

In the literature, drugs other then antimicrobials have been associated with thrombocytopenia, especially heparin [13,33]. Though, with an incidence of 0.1-0.5% [13], heparin-induced thrombocytopenia may have occurred in approximate 5 to 10 of the patients in our study. The ICU’s participating in our study used heparin products as part of routine care in immobilized patients irrespective of which of the two randomized arms the patient was allocated to and which type of antimicrobial regiment the patient received. Therefore, it is unlikely that unnoticed use of heparin is a relevant confounder that explains our findings. 

Our study has a number of shortcomings. As discussed above, several factors may be associated with the probability of using certain antimicrobials which may introduce bias due to confounding. Antimicrobials are often given in combinations related to type of infection. Using the randomized set-up gave us the unique opportunity to overcome this problem strengthening the clinical relevance of our findings relatively to the existing literature on this topic. Investigating the association between single agents and thrombocytopenia in the observational section of the analyses, we adjusted for infection status and site of randomization and hence minimized a potential difference in empiric treatment. We also performed stratified analyses on the subset of patients who did not present with severe sepsis/septic shock and assured consistency of the results across these strata. Furthermore, we did not include a model investigating continued vs. intermittent use of an agent. However, use of antimicrobials was fitted as a time-updated variables (i.e. current use), which made it possible to detect the effect on the platelet count while the patients were exposed to the specific agent.

 When investigate the single effect of ciprofloxacin, found to be the drug with the greatest association with thrombocytopenia, a model was created comparing current use of a drug with the same drug prescribed in combination with ciprofloxacin. This particular analysis made is possible to detect the single effect of ciprofloxacin. Although we cannot exclude the possibility that the confounding by indication bias was present, it was reassuring that similar differences between drugs were observed when using different approach to analyses.

## Conclusion

Exposing critically ill patients to waste amount of broad-spectrum antimicrobials as part of a large randomized control trial, did not suggest a major effect of antimicrobials in inducing absolute nor relative thrombocytopenia nor was the absolute platelet count affected. Use of ciprofloxacin, and to a lesser extent use of piperacillin/tazobactam, may singularly affect the platelet count slightly; however most episodes of thrombocytopenia among critically ill patients appears to be driven by other factors than antimicrobials, especially underlying severe infection. Thus, antimicrobial-induced thrombocytopenia may be of importance in the individual patient case, but it does not represent a significant problem in a more general population of critically ill patients. 

## Supporting Information

Figure S1 **Supplementary material. Flowchart displaying the progress through the analysis.**
(DOCX)

File S1 **Original study protocol from the The Procalcitonin and Survival Study (PASS).**
(DOC)

File S2 **Original statistical analysis plan.**
(DOCX)
