# Supplementary material for: The Potential of Antimicrobials to Induce Thrombocytopenia in Critically Ill Patients: Data from a Randomized Controlled Trial
Source: PLoS One. 2013 Nov 28;8(11):e81477. doi: 10.1371/journal.pone.0081477 (PMC3842947; doi:10.1371/journal.pone.0081477)
Supplement: Figure S1 — Supplementary material. Flowchart displaying the progress through the analysis. (DOCX) [file pone.0081477.s001.docx]

**Figure S1. Supplementary material. Flowchart displaying the progress through the study**
