# Supplementary material for: The Potential of Antimicrobials to Induce Thrombocytopenia in Critically Ill Patients: Data from a Randomized Controlled Trial
Source: PLoS One. 2013 Nov 28;8(11):e81477. doi: 10.1371/journal.pone.0081477 (PMC3842947; doi:10.1371/journal.pone.0081477)
Supplement: File S1 — Original study protocol from the The Procalcitonin and Survival Study (PASS). (DOC) [file pone.0081477.s002.doc]

**Protocol**

| **A randomised, single-blinded, multicentre trial to investigate if clinical management guided by daily standardised Procalcitonin measurements can reduce the mortality in critically ill patients**  **The Procalcitonin and Survival Study (PASS)** |
| --- |

**Version of protocol: 1.12**

**Date: 08. June 2005**

**Intensive Care Units from the following University Hospitals in Denmark will participate:**

Hvidovre Hospital, Bispebjerg Hospital, Amager Hospital, Herlev Hospital, Glostrup Hospital, Gentofte Hospital, Hillerød Sygehus

**Sponsor: Det Sundhedsvidenskabelige Forskningsråd (SVFR)**

**Protocol co-ordinator**

**Jens-Ulrik Stæhr Jensen**

H:S Hvidovre University Hospital

DK - 2650 Hvidovre

Denmark

Phone: +45 36 32 33 07

Fax: +45 36 47 33 40

E-mail: kirlusnej@tiscali.dk

**INVESTIGATOR PROTOCOL AGREEMENT PAGE**

*THIS AGREEMENT IS EQUIVALENT TO A “SIGNED PROTOCOL”*

**The PASS Trial**

Name and qualifications of investigator:

*Name of Investigator: __________________________________________________________*

*Post held: ___________________________________________________________________*

*Clinical Centre: _______________________________________________________________*

I agree:

• to assume responsibility for the proper conduct of the PASS Trial at this site.

• to conduct the trial in compliance with this protocol, any future amendments, and with any other trial conduct procedures provided.

• not to implement any deviations from or changes to the protocol without agreement from the sponsor and prior review and written approval from the Independent Ethics Committee (IEC), except where necessary to eliminate an immediate hazard to the subjects, or for administrative aspects of the trial (where permitted by all applicable regulatory requirements).

• that I am thoroughly familiar with the appropriate use of the Procalcitonin test and the interpretation of the test results, as described in this protocol, and any other information provided by the manufacturer of the test and by the PASS Coordinating centre.

• that I am aware of, and will comply with, ‘‘Good Clinical Practice’’ (ICH-GCP Guideline (CPMP/ICH/135/95, Directive 2001/20/EC)) and all applicable regulatory requirements.

• to ensure that all persons assisting me with the trial are adequately informed about the Procalcitonin test and interpretation and of their trial-related duties and functions as described in the protocol.

___________________________________________________ _________________

Signature of investigator Date

*One signed copy each to be held by the Investigator and PASS Co-ordinating centre.*

TABLE OF CONTENTS

PROTOCOL SUMMARY [5](#__RefHeading___Toc101760046)

1 TRIAL BACKGROUND AND RATIONALE [7](#__RefHeading___Toc101760047)

1.1 Background [7](#__RefHeading___Toc101760048)

1.2 Rationale - summary [8](#__RefHeading___Toc101760049)

1.3 Procalcitonin analysing methods [9](#__RefHeading___Toc101760050)

1.4 Rationale for a 24 h interval between blood sampling [10](#__RefHeading___Toc101760051)

1.5 Procalcitonin and immuno-compromised patients [10](#__RefHeading___Toc101760052)

1.6 Studies on Procalcitonin biology and bacterial infection [10](#__RefHeading___Toc101760053)

1.6.1 In vitro and animal studies [10](#__RefHeading___Toc101760054)

1.6.2 Human observational studies [11](#__RefHeading___Toc101760055)

1.6.3 Clinical trials [11](#__RefHeading___Toc101760056)

2 TRIAL OBJECTIVES AND ENDPOINTS [11](#__RefHeading___Toc101760057)

2.1 Trial Objectives [11](#__RefHeading___Toc101760058)

2.2 Primary Objectives [11](#__RefHeading___Toc101760059)

2.3 Secondary Objectives [11](#__RefHeading___Toc101760060)

2.4 Trial Endpoint(s) [12](#__RefHeading___Toc101760061)

3 INVESTIGATIONAL PLAN [13](#__RefHeading___Toc101760062)

3.1 Trial Design [13](#__RefHeading___Toc101760063)

3.2 Trial Population [14](#__RefHeading___Toc101760064)

3.2.1 Inclusion Criteria [14](#__RefHeading___Toc101760065)

3.2.2 Exclusion Criteria [15](#__RefHeading___Toc101760066)

3.3 Treatment During Trial [15](#__RefHeading___Toc101760067)

3.3.2 Change of PCT-guidance strategy during the trial [18](#__RefHeading___Toc101760068)

3.3.2.1 Randomised PCT-guided interventions [18](#__RefHeading___Toc101760069)

3.3.2.2 The non-PCT guided interventions [18](#__RefHeading___Toc101760070)

3.3.3 Antimicrobial Drugs and Dosages [18](#__RefHeading___Toc101760071)

3.3.3.1 Overdose and Toxicity [19](#__RefHeading___Toc101760072)

4 MEASUREMENTS AND EVALUATION [20](#__RefHeading___Toc101760073)

4.1 Time and Events Schedule [20](#__RefHeading___Toc101760074)

4.1.1 Pre-entry Evaluations [20](#__RefHeading___Toc101760075)

4.2 On Trial Evaluations [22](#__RefHeading___Toc101760076)

4.3 Trial drugs [24](#__RefHeading___Toc101760077)

4.3.1 Dosing Details [24](#__RefHeading___Toc101760078)

4.3.2 Collection of Blood Samples for Daily Analysis [24](#__RefHeading___Toc101760079)

5 DATA ANALYSIS METHODS [25](#__RefHeading___Toc101760080)

5.1 Sample Size Determination [25](#__RefHeading___Toc101760081)

5.2 General Considerations [25](#__RefHeading___Toc101760082)

5.2.1 Analysis Populations [25](#__RefHeading___Toc101760083)

5.2.2 Interim Analysis [26](#__RefHeading___Toc101760084)

5.2.3 Other Issues [26](#__RefHeading___Toc101760085)

5.3 Efficacy [26](#__RefHeading___Toc101760086)

5.3.1 Primary Efficacy Endpoint [26](#__RefHeading___Toc101760087)

5.3.2 Secondary Efficacy Endpoint(s) [26](#__RefHeading___Toc101760088)

5.3.2.1 Other mortality assessments [26](#__RefHeading___Toc101760089)

5.3.3 Combined evaluation of mortality / occurrence of serious bacterial infection while admitted to the ICU [27](#__RefHeading___Toc101760090)

5.4 Safety [28](#__RefHeading___Toc101760091)

6 ADVERSE EVENTS (AE) AND SERIOUS ADVERSE EVENTS (SAE) [28](#__RefHeading___Toc101760092)

6.1 Definition of an Adverse Event [28](#__RefHeading___Toc101760093)

6.1.2 Definition of a Serious Adverse Event [29](#__RefHeading___Toc101760094)

6.1.3 Protocol-specific Exemptions from the AE and SAE Definition [30](#__RefHeading___Toc101760095)

6.2 Clinical Laboratory Abnormalities and Other Abnormal Assessments as AEs and SAEs [31](#__RefHeading___Toc101760096)

6.3 Recording of the AEs and SAEs in the CRF [31](#__RefHeading___Toc101760097)

6.4 Documenting AEs and SAEs [31](#__RefHeading___Toc101760098)

6.5 Follow-up of AEs and SAEs [32](#__RefHeading___Toc101760099)

6.6 Time-lines for reporting of SAEs [32](#__RefHeading___Toc101760100)

7 TRIAL ADMINISTRATION [33](#__RefHeading___Toc101760101)

7.1 Data Collection [33](#__RefHeading___Toc101760102)

7.2 Regulatory and Ethical Considerations [34](#__RefHeading___Toc101760103)

7.2.1 Regulatory Authority Approval [34](#__RefHeading___Toc101760104)

7.2.2 Ethics Approval [34](#__RefHeading___Toc101760105)

7.2.3 Subject Informed Consent [34](#__RefHeading___Toc101760106)

7.3 Trial Monitoring [35](#__RefHeading___Toc101760107)

7.4 Quality Assurance [36](#__RefHeading___Toc101760108)

7.5 Trial and Site Closure [36](#__RefHeading___Toc101760109)

7.6 Records Retention [37](#__RefHeading___Toc101760110)

7.7 Information Disclosure and Inventions [37](#__RefHeading___Toc101760111)

7.7.1 Confidentiality [37](#__RefHeading___Toc101760112)

7.7.2 Publication [38](#__RefHeading___Toc101760113)

7.8 Indemnification and Compensation for Injury [38](#__RefHeading___Toc101760114)

8. REFERENCES [39](#__RefHeading___Toc101760115)

9. APPENDICES 43

Appendix 1: The Declaration of Helsinki 43

Appendix 2: Abbreviations [47](#__RefHeading___Toc101760116)

Appendix 3: Table of conversion factors for laboratory units [48](#__RefHeading___Toc101760117)

Appendix 4: Table of the used antibacterial and antifungal drugs 48

**A randomised, single blinded, multicentre trial to evaluate whether daily Procalcitonin measurements and immediate diagnostic and therapeutic response on abnormal values and day-to-day changes can reduce the mortality of critically ill patients in the Intensive Care Unit.**

**The Procalcitonin And Survival Study (PASS)**

# PROTOCOL SUMMARY

**Inclusion:**

Fulfilment of all of the following three criteria:

1. Male or female, aged > 18 years of age.
2. Admitted to the participating intensive care units (ICU) at following hospitals: Hvidovre Hospital, Bispebjerg Hospital, Amager Hospital, Herlev Hospital, Glostrup Hospital, Gentofte Hospital
3. 1) Ability to understand and provide written informed consent to participate in this trial,

**or**

2) Ability to understand and provide oral informed consent in presence of at least one impartial witness who should sign and personally date the consent form

**or**

3) The subjects legally acceptable representative can understand and provide written informed consent if the subject is not capable of this because of the present mental or physical condition of the subject.

**Exclusion:**

A subject will **NOT** be eligible for inclusion in this trial if any of the following criteria apply:

1. Subjects with known hyper-bilirubinaemia (>0.4 mg/ ml) or hypertriglyceridaemia (>10 g/l) since this can interfere with measurements. If subjects with unknown status on these points are included and have PCT measurements, the measuring-equipment will detect these conditions.
2. Subjects suffering from a blood disorder, where daily sampling of 7 ml of blood for maximally 28 days (210 ml distributed on 28 days) will be an inconvenience or a potential risk, which could compromise the safety of the subject.
3. Subjects who are pregnant or breast feeding

The *a priori* probability of surviving with the normal recommended diagnostics and treatment with the presently available means to detect infections and on the other hand the normal diagnostics and treatment together with daily Procalcitonin measurements and prompt clinical reaction should be equal.

**Randomisation**:

Two arms (1:1), n = 500 per arm:

Arm 1: Normal recommended diagnostics and treatment of infections in the intensive care unit (standard of care)

Arm 2: Normal recommended diagnostics and treatment of infections in the intensive care unit (standard of care) **and** Procalcitonin guided diagnostics and treatment of infection

**Primary Trial Objective:** To address whether daily Procalcitonin measurements and immediate diagnostic and therapeutic response on abnormal values and day-to-day changes can reduce the mortality of critically ill patients in the ICU.

**Trial registration days:** Intensive Care Unit admission day, running routine registration of examinations and blood tests, day of discharge or death, day 28 after admission, day 60, 90, 120 and 180 after discharge.

**Data collection:** The data collection will be simple and performed real time via fax.

# TRIAL BACKGROUND AND RATIONALE

## Background

###### Sepsis and mortality in the Intensive Care Unit

Sepsis remains a major cause of mortality in critically ill patients admitted to the Intensive Care Unit (ICU) ^1-2^. All-cause mortality during ICU admission ranges from 12.1% in non-infected patients to 43.9% in infected patients^3^. Patients who are discharged to other departments and later to their own home or an institution for rehabilitation, continue to have a high mortality (additionally 10-20%) for 20-30 days after ICU discharge^4-7^. Different explanations for this have been proposed. Among the most important are:

1. During ICU admission it becomes clear that further treatment lacks perspective for the patient (often chronical organ diseases and cancer diseases) and the patient is therefore discharged to the relevant department when discharge from the ICU is possible.
2. After discharge from the ICU the physical condition of the patient deteriorates because of a severe disease with a dismal prognosis and it is decided together with the patient and relatives that the patient should not be admitted to the ICU again.
3. Critically ill patients often have an immunological incompetence and therefore these patients are susceptible to serious infections. Additionally these infections often have an atypical course and thereby a delayed diagnosis. This immunological incompetence prevails some time after discharge from the ICU why the patient remains susceptible to infections for this period of time. There is a grave risk that these serious infections with an atypical course can be diagnosed late in the course and cause an increased risk of mortality for critically ill patients.

###### Procalcitonin and bacterial infections

In 1993 Assicot et al. reported that a high level of serum-Procalcitonin (PCT) was closely related to bacterial infection and seemingly correlated to the severity of the infection^8^. This finding has since been ascertained in many studies demonstrating high levels (2.0 ng/ml-50.0 ng/ml (-1500 ng/ml)) of PCT in patients with systemic bacterial infection, while low levels have consistently been found in patients with localised bacterial infections and viral infections^9-16^. Others have shown low PCT levels (and seldom up till maximally 3.0 ng/ml) in non-infected patients following surgery, trauma and myocardial infarction^10, 17-21^. Sensitivity and specificity for sepsis when PCT levels are above 5.0 ng/ml have been estimated to 80-90 % and 85-100%, respectively, in the largest of these studies.

The PCT level starts decreasing within 24 h after surgery, trauma and myocardial infarction in non-infected patients in contrast to the C-reactive protein, which has a peak level 36-72 h after these events^10-17-21^.

Consequently, bacterial infection is suspected if PCT is increasing 24 h after surgery, trauma or myocardial infarction.

###### Procalcitonin kinetics, biochemistry and cellular biology

PCT is a 13 kDa, 116 amino acid polypeptide, initially described as a pro-hormone of Calcitonin, a hormone in the calcium metabolism, which is produced in the medullary C-cells in the thyroid gland^22-24^. Recent studies have shown that the PCT variant, which is related to infection is produced in other tissues (liver, kidney, muscle, fat)^25-27^

Kinetic studies with healthy humans and baboons have shown a rapid release of PCT within 2-6 hours after injection of bacteria or bacterial endotoxin. This time to release is significantly shorter than that of C-reactive protein (8-24 h). The plasma half life of PCT is approximately 24 h. PCT measurements in healthy, uninfected volunteers has been shown very low levels (<0.05 ng/ml)^10,28-29^.

###### Procalcitonin-guided treatment and reduction in the use of antimicrobial agents

A recent study has demonstrated a reduced use of antimicrobial agents in patients with lower respiratory tract symptoms, when the treatment was guided by the initial PCT level^30^.

###### Procalcitonin and risk of mortality

We have shown that a PCT increase after reaching a level of 1.0 ng/ml is an independent predictor of mortality in critically ill patients. Patients who did not reach a PCT level above 1.0 ng/ml had an all cause mortality risk of 4.7% while admitted in the ICU, compared to an all cause mortality of 19.1% for the whole population of ICU patients. Patients who reached a PCT value above 1.0 ng/ml who had a decreasing PCT the next day had a mortality risk of 18.9%, but patients who had an increasing PCT level after reaching 1.0 ng/ml had a mortality risk of 32.7%. This increase in mortality risk was significant for the entire follow-up period of 90 days^31^.

The mortality risk increased for every day the PCT increased. Taking in mind the close relation between PCT levels and bacterial infection, a large part of this mortality increase is (when PCT is increasing), to the best of the existing knowledge, attributable to uncontrolled bacterial infections. This is supported by the findings of the European Sepsis Group^3^.

The rapid release of PCT to the blood stream (2-6 h), when infection is progressing, makes acute detection of ongoing serious infection possible, hereby potentially reducing mortality in critically ill patients if treatment is guided acutely by PCT measurements.

## Rationale - summary

Sepsis and complications to sepsis are major causes of mortality in critically ill patients^1-2^. Rapid treatment of sepsis is of crucial importance for survival of patients. In the ICU, the infectious status of the patient is often difficult to assess because symptoms cannot be expressed (unconscious or sedated patients) and signs may present atypically because of immunologic incompetence and masking by the drugs given and thermo-therapy. Biological and biochemical markers of inflammation (WBC, C-reactive protein) may often be influenced by other parameters than infection, such as: trauma, surgery, other types of inflammation such as rheumatoid diseases (C-reactive protein) and gluco-corticosteroid treatment (WBC), and may be unacceptably slowly released after progression of an infection^32-33^. At the same time, lack of a relevant antimicrobial therapy in an early course of infection may be fatal for the patient.

For these reasons, in the clinical setting, it is often necessary to initiate or adjust antimicrobial therapy on an unsure ground and the relevant therapy may in some situations be delayed for important hours or even days. Specific and rapid markers of bacterial infection have been sought for use in the ICU. Mortality in critically ill patients increases gravely when Procalcitonin levels increase from day to day^31^. Low PCT levels have been shown to effectively rule out sepsis^12^.

However, no randomised controlled trials have been conducted to show if mortality in critically ill patients can be reduced by using a strategy of daily standardised Procalcitonin measurements as an early detector of serious bacterial infection. Therefore evidence is presently not sufficient to introduce daily consecutive Procalcitonin measurements to guide the diagnostic and therapeutic management of patients admitted to the ICU .

The rationale for this trial is to assess the ability of daily Procalcitonin measurements to reduce the mortality of critically ill patients.

## Procalcitonin analysing methods

There are four commercially available analysing methods for measuring blood levels of Procalcitonin, one semi-quantitative and three quantitative. Two of these are described below, the oldest and most used test, *LUMITEST ® BRAHMS /BRAHMS PCT LIA,* and a newer fully automated test with a higher sensitivity, *KRYPTOR® PCT BRAHMS.* KRYPTOR® PCT BRAHMS will be used for all Procalcitonin analyses in this study^34^.

- - 1. **LUMITEST ® BRAHMS /BRAHMS PCT LIA**

The oldest and so far most used quantitative test is LUMITEST ® BRAHMS /BRAHMS PCT LIA.

Analysis is made by a ”sandwich” luminiscens immuno-assay with an anti-catacalcin coated tube:

Anti-**Catacalcin** binds catacalcin in the patient sample and is hereby immobilised (catacalcin could otherwise interfere with the analysis).

Anti-**Calcitonin** antibody is marked with a luminescent a*cridin-*derivative.

H_2_O_2_ and NaOH are added and these react with the *acridin-*derivative which leads to the formation of *acridon* and this process is accompanied by transmission of light. The quantity of this light is proportional to the Procalcitonin concentration in the sample.

We have found a coefficient of variation (CV) in the measuring interval between 0.1 ng/ml-1.0 ng/ml of 0.09-0.83 for this test. At PCT levels above 1.0 ng/ml, we found CV´s of 0.008-0.065 (range)^37^.

The manufacturer claims a *functional assay sensitivity* (CV<0.2) of 0.3 ng/ml.

- - 1. **KRYPTOR® PCT BRAHMS**

A new, and according to the manufacturer, more precise assay is the fully automated KRYPTOR® PCT BRAHMS. Procalcitonin is analysed using the analysing machine KRYPTOR® and fluids and utensils from the company BRAHMS diagnostica, Berlin. KRYPTOR® uses TRACE technology (Time Resolved Amplified Cryptate Emission), which is a non-radiating transmission of energy. The transmission happens between two flourescent compounds: Europium Cryptate (donor) and XL665 (acceptor). While the antigen-antibody complex is formed, a signal is measured.

The functional assay sensitivity (CV< 0.2) is according to the manufacturer 0.06 ng/ml for the KRYPTOR ® test. In the relevant clinical interval (which has not quite been defined yet) the CV is 0.02-0.03 (product information).

- Studies concerning Procalcitonin have so far mainly been using LUMITEST ® BRAHMS /BRAHMS PCT LIA.

## Rationale for a 24 h interval between blood sampling

Several studies have shown a half-life of Procalcitonin of 20-30 hours and Procalcitonin levels increase 2-6 h after bacterial products are presented in the blood stream ^10,28-29, 35^. An important exception to this is patients suffering from severe uraemia, where the Procalcitonin half-life is prolonged, but it has been demonstrated, that Procalcitonin is removed by dialysis^35^. Studies concerning Procalcitonin and surgery have shown, that the Procalcitonin blood level is on a decreasing curve 24 h after major thoracic and abdominal surgery, except in infected patients^17-21^. In conclusion, a Procalcitonin level which is increasing 24 h after a therapy shift or after surgery suggests progression of infection.

## Procalcitonin and immuno-compromised patients

Markers and mediators of inflammation and infection are often dependent on a functioning immune system, which is able to produce the substance measured, e.g. WBC, TNF, different interleukins^10,15,16, 36^. It has been established that Procalcitonin is not dependent on blood cells and their mediators, and Procalcitonin is mainly produced by tissues like liver, kidney, muscle and fat^25-28^. In concordance with this, studies investigating Procalcitonin in neutropenic patients have found results comparable to those for immuno-competent patients^36-41^. A few studies regarding neutropenic patients that compared PCT levels to positive blood cultures have found a low sensitivity of the test for bacteriemia, but these studies lack clear definitions of virulence of different micro-organisms (e.g. Coagulase negative staphylococci vs. Gram negative rods) in their study designs^40^.

## Studies on Procalcitonin biology and bacterial infection

#### In vitro and animal studies

In vitro studies have shown Procalcitonin to be an inducer of albumin synthesis in rat liver tissue measured on mRNA and protein synthesis. This was found to be opposite to TNFα and IL-6, these substances lowering albumin synthesis^42^. In a study of sepsis in baboons, low PCT was found in non-infected subjects and high PCT in infected subjects, and PCT blood levels started increasing after 2 hours^10^. In another baboon model Procalcitonin incompetence was shown in an anhepatic subject^28^.

In a study of burn wound and Pseudomonas aeruginosa septicaemia in rats, a high correlation between endotoxin levels and PCT in blood was found^43^.

#### Human observational studies

Most of the present knowledge on Procalcitonin has been established by observational studies. Key-references are mentioned in paragraph 1.1 and 1.2

### Clinical trials

The only clinical trial so far randomising for PCT-guided treatment (n=119+124), assessed the ability of this clinical strategy to reduce use of antimicrobial therapy in patients with suspected lower respiratory tract infection. A Relative Risk of 0.49 [95% CI 0.44-0.55] for antibiotic exposure was demonstrated, without any significant difference in culture growth from patient samples, quality of life, mortality, inflammatory parameters (temperature, C-reactive protein, WBC), number of days admitted and need for stay in intensive care unit. The study was designed to detect a 30 % difference with 95% stringency. However some of the mentioned endpoints do not occur in all patients, and in these cases (mortality, need for stay in ICU) it may be false to conclude, that there is no difference between groups within the chosen 30 % limit^30^.

A very small study (n=12+13=25) has tried to investigate empiric prophylaxis with fluor-quinolone Ofloxacin in patients with abdominal aortic aneurism. However the sample size of this study does not justify any conclusions on this issue^44^.

# TRIAL OBJECTIVES AND ENDPOINTS

## Trial Objectives

## Primary Objectives

To address whether immediate diagnostic and therapeutic initiatives guided by abnormal high and increasing values of Procalcitonin measured daily can reduce the mortality of critically ill patients in the ICU.

## Secondary Objectives

- 1. To determine mortality of ICU patients at discharge from the ICU, at day 60,90, 120 and 180.
  2. To determine differences in prescription of antimicrobial therapy in the two arms.
  3. To determine the frequency of patients with complications to infection in the two arms, defined as; sepsis, severe sepsis, septic shock, disseminated intravascular coagulation, multi-organ dysfunction syndrome (MODS), coma (Glasgow Coma Scale), hypotension, respiratory insufficiency (ventilator treatment need), liver insufficiency, acute uremia (three times increase in baseline creatinine).
  4. APACHE II score
  5. Accumulated PCT increases over time
  6. To determine the number of diagnostic image procedures per day after enrolment in the trial in the two arms
  7. To determine the number of non-routine microbiological samples taken per day after enrolment in the trial in the two arms
  8. To determine the number of surgical procedures per day after enrolment in the trial in the two arms
  9. To determine the time to the first change in antimicrobial chemotherapy after admittance to the ICU in the two arms

## Trial Endpoint(s)

***Primary:***

Mortality at day 28 after admission to the ICU.

***Secondary:***

1. Mortality while admitted to the ICU, Mortality at day 60, 90 and 180 after admission to the ICU
2. Defined day doses of antimicrobial therapy in each arm
3. Occurrence of sepsis, severe sepsis, septic shock, DIC. Assessment of Glasgow Coma Scale, measurement of Blood Pressure (systolic blood pressure < 90), days with artificial ventilation, Factor 2-7-9 < 0.7, creatinine (increase factor 3 from baseline), MODS.
4. APACHE II score daily (Temperature, Mean Arterial Pressure, Heart Rate, Respiratory Rate, FIO_2_, HCO_3_^-^ , pH (arterial), Se- Na^+^, K^+^, Creatinine, Haematocrite, White Blood Count+ differential count, Glasgow Coma Scale).
5. AUC_Procalcitonin_ for the Procalcitonin-measuring group and for the control group.
6. Number of diagnostic images after admission to the ICU.
7. Number of non-routine microbiological sample taken after admittance to the ICU.
8. Number of surgical procedures during the trial
9. Time to the first change in antimicrobial chemotherapy after admittance to the ICU

# INVESTIGATIONAL PLAN

## Trial Design

- - 1. **Intervention**

This is a randomised, single-blinded multicentre trial.

Approximately 1000 subjects admitted to an ICU in the participating University hospitals will be included. All patients included will receive the the standard recommended diagnostic and therapeutic procedures mandated at the particular ICU. Additionally, the patients will be randomised for:

1. No PCT guided diagnostics and treatment (i.e. the standard-of-care / **control arm**).

**Or**

1. Daily PCT measurements and protocol-specified additional diagnostic and/or therapeutic interventions guided by the PCT levels observed. High or increasing PCT levels will mandate such interventions (see section 3.3.1 for details of interventions)(the **PCT intervention arm**)
   - 1. **Randomisation**

The randomisation is performed by the PASS study centre and is stratified according to site,, age and initial Acute Physiology And Chronic Health Evaluation II (APACHE II) score. For patients randomised to the PCT intervention arm, daily PCT levels are communicated to the team responsible for the clinical management together with a recommendation of what interventions the investigator team is expected to initiate based on the PCT measurement. In the control arm, blood samples for PCT will be analysed simultaneously with samples from the PCT intervention arm, but results of these PCT analyses will remain blinded for the investigators until the study has been completed. The PCT measurements will be conducted daily as long as the patient is admitted to the ICU, but maximally 28 days from time of enrolment in this study. While patients remain in the hospital, and after discharge from the ICU, samples will be collected for PCT determination but the samples will not be analysed real-time and hence the results will not be used to guide interventions outside the ICU, except if requested by the ICU investigator in conjunction with the discharge of the patient. Patients transferred from one ICU to another ICU, will remain in the trial provided that the receiving ICU also participates in this trial.

## Trial Population

### Inclusion Criteria

A subject will be eligible for inclusion in this trial only if all of the following criteria apply:

1. Male or female, aged > 18 years of age.
2. Admitted to the participating intensive care units. Patients should be included within 24 h. If a patient has not been included at this time, this patient cannot be included in the present admittance.
3. Subjects should in the investigator’s opinion be likely to be admitted to the ICU for more than 24 h. Subjects should not be likely (<10%) to die or be discharged in this period of time
4. Ability to understand and provide written informed consent to participate in this trial,

**or**

Ability to understand and provide oral informed consent in presence of at least one impartial witness who should sign and personally date the consent form

**or**

The subjects legally acceptable representative can understand and provide written informed consent if the subject is not capable of this because of the present mental or physical condition of the subject.

### Exclusion Criteria

A subject will **NOT** be eligible for inclusion in this trial if any of the following criteria apply:

1. Subjects with known hyper-bilirubinaemia (>0.4 mg/ ml) or hypertriglyceridaemia (>10 g/l) since this can interfere with measurements. If subjects with unknown status on these points are included and have PCT measurements, the measuring-equipment will detect these conditions.
2. Subjects suffering from a blood disorder, where daily sampling of 7 ml of blood for maximally 28 days (210 ml distributed on 28 days) will be an inconvenience or a potential risk, which could compromise the safety of the subject.

## Treatment During Trial

The aim of the PCT guided treatment is to reduce time to relevant treatment of a serious infection and thereby to reduce the mortality. All subjects will receive the standard-of-care evaluations and therapeutic interventions recommended in the ICU at which the patient is admitted to. Subjects in the PCT measurement group will additionally receive expanded diagnostics and treatment should the PCT levels be found to high and/or increasing (see section 3.3.1 for definitions).

Access to results of PCT measurements of any kind (semi-quantitative or quantitative) at any time in the study period is not allowed for patients randomised to the control arm.

The PASS study group in collaboration with the PASS Steering Committee, will issue guidelines for the composition of the interventions that a high or increasing PCT level would mandate. Some variation between sites is acceptable, whereas all patients within a given ICU should follow that ICU’s guidelines. The guidelines will be updated when new information becomes available. In the guidelines, there may be several alternatives indicated for a given situation. The investigator is not mandated to follow the guidelines.

- - 1. **Procalcitonin levels and diagnostic and therapeutic consequenses**

The situation mandating additional interventions in the the PCT intervention arm is based on the following criteria:

- PCT levels ≥ 1.00 ng/ml

**and**

- The PCT level increases one day to the next or has an irrelevant decrease of < 10%

The daily assessment of PCT guided interventions will be as follows:

- Subjects with PCT levels ≥ 1.00 ng/ml based on the first determination after enrolment into the study will follow the principles for interventions as detailed below.
- Subjects with PCT levels ≥ 1.00 ng/ml and with a day (n) to day (n+1) PCT increase or a decrease of < 10% (irrelevant decrease) will follow the principles for interventions as detailed below.
  - Microbiology: blood cultures, airway cultures, urine cultures and samples from any other suspected foci.
  - Diagnostic imaging: one or more of the following: Chest X-ray, Ultra-sonic examination of suspected focus, Computerised Tomography of relevant areas, Magnetic Resonance imaging of relevant areas, other imaging techniques.
  - Surgical drainage of possible un-drained foci
  - Antimicrobial therapy expansion. Treatment will be guided by any relevant findings: microbial or diagnostic imaging, or other findings. If focus and micro organism of infection is not clear steps will be:

1) Empirical sepsis treatment

2) Empirical sepsis treatment with anaerobic and gram positive coverage

3) Empirical sepsis treatment with anaerobic and gram positive coverage and/ or fungal treatment

- Subjects with PCT levels < 1.00 ng/ml will continue to receive standard-of-care
- Subjects with PCT levels ≥ 1.00 ng/ml and with a day-to-day PCT **decrease** of ≥ 10% will continue to receive standard-of-care.

Precise guidelines for this (antimicrobial) treatment will be made specifically for every ICU in concordance with the local choices regarding antimicrobial agents. For PCT guided diagnostics and treatment algorithm, see Diagram 1:

**All ICU patients**

**No Procalcitonin measurements,**

*Standard of Care*

**Procalcitonin measurements (daily),**

The *Standard of Care* and additionally PCT guided diagnostics and treatment.

**PCT≥1.0 ng/ml**

**and**

**ΔPCT: Negative (≥10% decrease/24 h):**

- Continue ongoing surgical treatment and antimicrobial chemotherapy
- Adjustment of antimicrobial therapy according to relevant findings
- Antimicrobial therapy cannot be discontinued before PCT has been decreasing for ≥ 72 h or PCT < 1.0 ng/ml.

Relevant PCT decrease to <1.0 ng/ml

**PCT≥1.0 ng/ml**

**and**

**ΔPCT: Positive or not relevantly decreasing (<10%decrease/24h):**

- Culture samples from blood, urine, airways and any other suspected foci
- Acute diagnostic imaging if focus is not known
- Expansion of antimicrobial spectrum for every day PCT remains increasing (or not relevantly decreasing).
  - If no present antimicrobial treatment: Empirical sepsis treatment.
  - If subject is already in empirical sepsis treatment, spectrum is broadened with anaerobic/ gram positive/ fungal coverage according to the most likely microbial etiology.

**PCT <1.0 ng/ml:**

- No further investigations based on PCT changes
- Already defined foci are treated according to the recommended guidelines of the ICU.

**PCT <1.0 ng/ml:**

- No further investigations based on PCT changes
- Already defined foci are treated according to the recommended guidelines of the ICU.

Continued increase or not relevant decrease

Relevant decrease in PCT level, but not yet to <1.0 ng/ml

- **Antimicrobial treatment is NOT to be discontinued if PCT is increasing and > 1.0 ng/ml**
- **When treatment of infection is relevant, PCT normally decreases in less than 18 h. If PCT is still not decreasing at the next-coming measurement after a therapy shift, a new (expanded) strategy is to be instituted**

### Change of PCT-guidance strategy during the trial

#### Randomised PCT-guided interventions

Subjects may **discontinue** the interventions initiated on the basis of PCT measurements only in case the benefit: risk ratio for these interventions is not acceptable to the treating physician. The specific concern will be collected.

#### The non-PCT guided interventions

The recommended interventions based on other information than PCT measurements should always be instituted and continued when relevant from a clinical judgement.

### Antimicrobial Drugs and Dosages

All antimicrobial drugs prescribed on basis of an increasing PCT must be prescribed by the investigator or an intensive care physician, who has been sufficiently instructed in all aspects of the trial. The investigator must check for possible drug-drug interactions between any of the drugs prescribed guided by PCT changes and other agents that may be metabolised via the same enzyme systems or organs. To assist the investigator, information on this topic is included in the Manual of Operational Procedures. Also, the product label of each drug prescribed should be reviewed.

General principles that will be followed regarding antimicrobial therapy of sepsis are:

- Antimicrobial agents are prescribed, when possible, according to the resistance pattern of the causative microorganism.
- When the causative microorganism is not known, antimicrobial agents are prescribed according to knowledge of which microorganisms normally and possibly infect the suspected focus.
- When neither the microorganism nor the focus of infection is known, one or more broad spectrum antimicrobial agents are selected. If the effect is not sufficient, the spectrum of the used antimicrobial agents is additionally expanded, often with anaerobic active agents, gram positive active agents and antifungal agents. Conversely, if the effect is sufficient, the spectrum of used antimicrobial agents is narrowed according to knowledge of focus and causative microorganism.
- In empiric sepsis treatment, a combination of a ß-lactam/ Carbapenem + a fluor-quinolone is chosen if not contra indicated in the specific subject. This treatment can be supplemented with nitroimidazoles, glycopeptides, oxazolidinones and azoles. More specific treatment regimes are initiated and guided by findings regarding the causative microorganism and/or focus of infection.

Dosages of antibiotics are decided according to the recommendations of the specific ICU.

The toxicity management guidelines detailed below refer to all components of the antimicrobial treatment used in the trial.

#### Overdose and Toxicity

Antimicrobial agents may be interrupted because of the development of adverse events (AEs, see section 6.1 for definitions) at the discretion of the investigator and according to the severity of the AE. The dose of all antimicrobial drugs may be reduced, interrupted or reintroduced according to standard practice at the time, and depending on the severity of the AE.

Subjects who require a dose modification should be re-evaluated on a daily basis.

The investigator is responsible for taking appropriate precautions to ensure that the risk of developing toxicity is minimised, that the subject is monitored for the development of toxicity, and if such toxicities do occur, take appropriate action to minimise their effects.

# MEASUREMENTS AND EVALUATION

## Time and Events Schedule

A flow chart showing the timing of trial procedures (Clinical and Laboratory) is shown in Table 1.

An initial pre-entry (screening) assessment for eligibility will be performed as soon as possible after the patient is admitted to the ICU. The patient should be randomised no later than 24 hours after the time of admission. Evaluations will then be carried out at entry (Day 1), and thereafter daily as long as the patients remains in the ICU. After discharge, the course of disease is collected in less detail and the survival status determined day 28, 60, 90 and 180 after enrolment in the trial.

### Pre-entry Evaluations

The site must obtain subject consent in the form of a written informed consent form prior to the initiation of **any** pre-entry procedures as outlined in this protocol. The consent form must be approved by the IEC of each participating site.

The pre-entry evaluation will be conducted the first day of the trial by an investigator in the ICU and will include an evaluation of whether the patient fulfils the requirements for enrolment in this trial (see section 3.2.2 and 3.2.3.

Subjects who fail to meet the entry criteria may not be re-screened for this protocol until 28 days after the failed pre-entry evaluation. Hence, enrolment of such patients will require that the patient is re-admitted to the ICU after at least 7 days outside of the ICU after the time of the first screening.

- - 1. **Baseline (Day 1) Evaluations**

The following evaluations should be performed at baseline (Day 1):

Note: For this trial, Baseline (Day 1) is defined as the day on which the subject has his/her first blood sample for PCT measurement. The following data are to be collected on day 1:

- Demography including date of birth, weight, height, and indication for admittance to the ICU
- Infections found in the subject in this hospital admission prior to admittance to the ICU.
- Present infection focus/ etiologic microorganism
- APACHE II score (Temperature, Mean Arterial Pressure, Heart Rate, Respiratory Rate, FIO_2_, HCO_3_^-^ , pH (arterial), Se- Na^+^, K^+^, Creatinine, Haematocrite, White Blood Count+ differential count, Glasgow Coma Scale)
- Current medical conditions
- Pre-admittance daily function and health state:

Professional career: 1) Student, 2) Part time work, 3) Full time work,

4) Early retirement, 5) Retired

Health: 1) Congenital handicapped, 2) Acquired handicap, 3) Chronic disabling disease, 4) Chronic non-disabling disease, 5) Healthy

Self-supportance: 1) Lives in nursing home, 2) Lives in a flat connected to a nursing home, 3) Own home with external help ≥ once / day, 4) Own home with external help < once daily, 5) Own home, no help required

Hospital need: 1) ≥ 3 months admitted to a hospital/ last year, 2) 1-3 months admitted to a hospital/ last year 3) 1-30 days admitted/ last year, 4) No admissions, ambulatory visits ≥ 6/ last year, 5) No admissions, ambulatory visits 1-5/ last year, 6) No admissions, No ambulatory visits/ last year

- Adverse events/ other complications to treatment given in this hospital admission (ongoing clinical conditions at Day 1 shall be recorded in the “Adverse Event and Medical Condition Form” of the CRF at this time, regardless of the fact that such conditions may not subsequently be found to fulfil the definitions for an adverse event (see section 6.1))
- Haematology: *haemoglobin, platelet count (WBC count mentioned as part of APACHE II)*
- Clinical chemistry: Albumin, Bilirubin, Factor 2-7-9, Alanin Amino Transferase (ALAT)/ Aspartate Amino Transferase (ASAT), Alcaline Phosphatase, Creatinine, Carbamide, Na^+^, K^+^, Phosphate, Ca^2+^, C-reactive protein (some are also mentioned as part of APACHE II).
- Baseline PCT

The daily PCT determination is done real-time at the Department of Clinical Biochemical Department, Hvidovre Hospital, using the EC-approved measuring instruments and reagents. For each subject, the same methodology should be used throughout the trial period. The KRYPTOR® PCT BRAHMS sensitive assay is the accepted standard assay. Other licensed assays may be used instead if judged by the PASS steering committee to have a comparable performance compared to the indicated assay.

## On Trial Evaluations

On trial assessments will be completed at the following time-points unless otherwise specified:

While admitted to the ICU, the following information will be registered unless specified otherwise:

**Daily while patient is admitted to the ICU:**

- Clinical signs of new (nosocomial) infections
- Microbiological or radiological evidence of new (nosocomial) infection
- Defined Day Doses of antimicrobial chemotherapy
- APACHE II score (Temperature, Mean Arterial Pressure, Heart Rate, Respiratory Rate, FIO_2_, HCO_3_^-^ , pH (arterial), Se- Na^+^, K^+^, Creatinine, Haematocrite, White Blood Count+ differential count, Glasgow Coma Scale)
- Occurrence of sepsis, severe sepsis, septic shock, DIC. Assessment of Glasgow Coma Scale, measurement of Blood Pressure (systolic blood pressure < 90), days with artificial ventilation, Factor 2-7-9 < 0.7, creatinine (increase factor 3 from baseline), MODS.
- Adverse events/ other complications to treatment given in the ICU (ongoing clinical conditions at Day 1 shall be recorded in the “Adverse Event and Medical Condition Form” of the CRF at this time, regardless of the fact that such conditions may not subsequently be found to fulfil the definitions for an adverse event (see section 6.1))
- Haematology: haemoglobin, platelet count WBC (WBC count also mentioned as part of APACHE II)
- Clinical chemistry: Albumin, Bilirubin, Factor 2-7-9, Alanin Amino Transferase (ALAT)/ Aspartate Amino Transferase (ASAT), Alcaline Phosphatase, Creatinine, Carbamide, Na^+^, K^+^, Phosphate, Ca^2+^, C-reactive protein (some are also mentioned as part of APACHE II).
- Blood sample for PCT determination
- Diagnostic imaging procedures performed
- Non-routine microbiological sample taken
- Surgical procedures performed
- Change in antimicrobial chemotherapy

**At the day of discharge from ICU or day of death or later:**

- Mortality and time of death, and the cause hereof
- AUC_Procalcitonin_ (at discharge from the ICU) (will remain blinded in the control arm)
- Discharge and post-discharge daily function and health state (obtained on day 30 and 180):

Professional career: 1) Student, 2) Part time work, 3) Full time work,

4) Early retirement, 5) Retired

Health: 1) Congenital handicapped, 2) Acquired handicap, 3) Chronic disabling disease, 4) Chronic non-disabling disease, 5) Healthy

Self-supportance: 1) Lives in nursing home, 2) Lives in a flat connected to a nursing home, 3) Own home with external help ≥ once / day, 4) Own home with external help < once daily, 5) Own home, no help required.

Hospital need: 1) ≥ 3 months admitted to a hospital/ last year, 2) 1-3 months admitted to a hospital/ last year 3) 1-30 days admitted/ last year, 4) No admissions, ambulatory visits ≥ 6/ last year, 5) No admissions, ambulatory visits 1-5/ last year, 6) No admissions, No ambulatory visits/ last year

**After discharge from ICU while patient is still admitted to hospital**

- Clinical signs of new (nosocomial) infections
- Microbiological or radiological evidence of new (nosocomial) infection
- Defined Day Doses of antimicrobial chemotherapy
- Current medical conditions (including acute organ failures)
- Diagnostic imaging procedures performed
- Surgical procedures performed
- Blood sample for PCT determination – done daily

## Trial drugs

Drugs prescribed on basis of PCT levels and changes belong to following categories: Antibacterial chemotherapeutics and Antifungal chemotherapeutics. Drugs from these categories will also be prescribed for the control group (and in patients not included in the trial), when indicated from other findings than level/change of PCT. An exhaustive list of drugs, used in the participating ICU´s (and thereby also in the trial subjects and controls) is given in appendix

### Dosing Details

The following details on dosing of all prescribed antimicrobials during the study period must be recorded in the “Medication form” in the CRF.

- Date of initial therapy
- Dose at each dosing change, together with reason for change
- Date of last dose of each agent
- Reason for discontinuation
- Date of resumption of therapy

### Collection of Blood Samples for Daily Analysis

Plasma from the PCT group and the control group will be collected early each morning (01.00 a.m.-06.00 a.m.) and will be transported to the Department of Clinical Microbiology Hvidovre Hospital, DK-2650 Hvidovre (or other laboratories, that can provide a PCT analysis real-time and with an analysing method which is approved by the PASS coordinating centre) and analysed immediately hereafter. The results from this analysis will be communicated by FAX to the Intensive Care Units for patients randomised to the PCT intervention arm or concealed for patients randomised to the control arm. Remaining material for the blood samples will hereafter be frozen for later analysis of other biochemical, biological and genetic markers (-80^o^C). Once the trial has been completed, the coupling of these samples to person-identifiers will be broken, and hence subsequent analyses done without any possibility to connect the results to individual persons involved in the trial. For detailed instructions regarding the collection, labelling, processing and transport of samples, see the Manual of Operational Procedures.

It is the responsibility of the investigator (to be assisted by the courier service and PASS coordinating office) to ensure that all trial samples for transport are appropriately handled, packed and transported.

# DATA ANALYSIS METHODS

## Sample Size Determination

The trial will randomise (1:1) 1,000 subjects into two treatment arms:

1: Control arm

2: The PCT guided intervention arm

With a sample size of 500 per group and an assumed mortality rate of 25% in the control group and 17.5 % in the PCT group there will be 80% probability that a negative result (Confirming the Null Hypothesis) is true. At the same time there will be < 5% probability of falsely declaring the alternative hypothesis correct. [Power 80%, stringency 5%]. Sample Size calculations via Dept. of Statistics, UCLA, California, USA.

## General Considerations

### Analysis Populations

The primary population for analyses of the efficacy and safety data will be the intention to treat population, including all randomised subjects who have at least one blood sample made for PCT measurements.

Response to PCT guided diagnostic and therapeutic interventions will also be investigated descriptively by summary statistics for various sub-groups, e.g. gender, other demographic variables, Baseline APACHE II score, and pre-admittance health assessment.

### Interim Analysis

Safety and efficacy data will be reviewed when 250, 500 and 750 subjects have completed the trial period (until discharge from the hospital or death, maximally 28 days), or at least every 6 th month, and assessments will be made by an independent Data and Safety Monitoring Board (DSMB). A cut-off date will be specified at this point and all treatment failure and adverse event data before this date will be used.

The Peto method of repeated significance testing will be used to test for treatment difference and a p-value of 0.001 will be used as the significance level at the interim analysis, giving a significance level of 0.05 for the final analysis once all patients have completed the trial.

Stopping the trial will not be based purely on a statistical decision but also on the recommendation of the DSMB.

### Other Issues

All subjects will remain in the trial and be followed-up until day 180.

## Efficacy

### Primary Efficacy Endpoint

The primary efficacy analysis will be the comparison of the two treatment groups with respect to the incidence of mortality within 28 days after enrolment in the trial. Mortality is defined as all-cause mortality. Subjects not followed for the entire duration of the trial (i.e. lost to follow-up) will be counted as survivors. Very few patients will be lost to follow up for the primary endpoint, because of the Danish Central Person Register (CPR), where all deaths in Denmark are registered. Only subjects who permanently move their address to another country within 30 days after ICU admission can be lost to follow-up. The stratified log-rank test and Kaplan Meier estimates will be used.

### Secondary Efficacy Endpoint(s)

#### Other mortality assessments

The proportion of subjects, who survive to different points of time (at discharge, after 60, 90 and 180 days, counting after ICU admission). The log rank test and Kaplan-Meier estimates will be used. Differences in proportions of survivors will be assessed using the Mantel-Haenzel Chi Square test and Wilcoxon test. Subjects with missing mortality data will be classified as survivors.

- - - 1. **Other parameters than mortality**
- Defined day doses of antimicrobial therapy in each arm
- Occurrence of sepsis, severe sepsis, septic shock, DIC. Assessment of Glasgow Coma Scale, measurement of Blood Pressure (systolic blood pressure < 90), days with artificial ventilation, Factor 2-7-9 < 0.7, creatinine (increase factor 3 from baseline), MODS.
- APACHE II score daily (Temperature, Mean Arterial Pressure, Heart Rate, Respiratory Rate, FIO_2_, HCO_3_^-^ , pH (arterial), Se- Na^+^, K^+^, Creatinine, Haematocrite, White Blood Count+ differential count, Glasgow Coma Scale).
- AUC_Procalcitonin_ for the Procalcitonin-measuring group and for the control group.
- Number of diagnostic images after admission to the ICU.
- Number of non-routine microbiological sample taken after admittance to the ICU.
- Number of surgical procedures during the trial
- Time to the first change in antimicrobial chemotherapy after admittance to the ICU
- Occurrence of new clinically, microbiologically or radiologically diagnosed infections while admitted to the ICU
- Discharge and post-discharge daily function and health state

For endpoints that have normally distributed numbers, t-test will be used in assessment of statistical significance. If not normally distributed, Mantel-Haenzel Chi Square test and the Wilcoxon test, will be used.

Exploratory analysis of adjustments for possible confounders present at baseline for the analysis presented above will be performed using Cox proportional hazards and Logistic regression modelling (as appropriate).

### Combined evaluation of mortality / occurrence of serious bacterial infection while admitted to the ICU

The proportion of patients who die during the trial period or who experience occurrence of a serious bacterial infection (sepsis, severe sepsis, septic shock, Disseminated Intravascular Coagulation (DIC) or Multi Organ Dysfunction Syndrome (MODS) (which ever came first) as a function of time since trial initiation. In this analysis, patients discontinuing the randomised treatment for other reasons before having failed in this analysis will be censored from the time of discontinuation.

## Safety

Adverse events will be tabulated by treatment group, maximum intensity, attributability to various antimicrobial agents and by seriousness. Treatment related adverse events that lead the subject to prematurely discontinue one or more of the originally prescribed antimicrobial agents will also be summarised.

Clinical chemistry and haematology results will be presented by summary statistics and quartile plots of measured results. Change from baseline for these results will also be presented. Baseline is defined as the laboratory data collected at Day 1 (before the first blood sample for PCT analysis). Subjects must have both a baseline and an “on treatment” measurement to be included in the change from baseline analysis.

Treatment emergent toxicity grades will be presented for each graded laboratory parameter by treatment group. A graded toxicity is considered treatment emergent if it develops or increases in intensity, post Day 1. Treatments will include established and approved antimicrobial treatments, which are already used daily in the participating ICU´s.

Concurrent medications and blood products will be summarised by randomised treatment group.

# ADVERSE EVENTS (AE) AND SERIOUS ADVERSE EVENTS (SAE)

No new treatments are introduced in this study. All adverse events recorded in the study therefore relate to events occurring from established and approved antimicrobial treatments. All patients receive "standard of care". Patients with abnormal PCT values will receive a supplementary treatment with established antimicrobial therapy. No patients will be withheld a treatment, they should elsehow have received. The investigator is responsible for the detection and documentation of events meeting the definition of an AE or SAE as as detailed in this section of the protocol.

## Definition of an Adverse Event

An adverse event (AE) is defined as any untoward medical occurrence in a subject or clinical investigation subject administered a pharmaceutical product which does not necessarily have a causal relationship with this treatment. An AE can therefore be any unfavourable and unintended sign (that could include a clinically significant abnormal laboratory finding), symptom, or disease temporally associated with the use of a medicinal product, whether or not considered related to the medicinal product.

AEs (including SAEs, see section 6.1.2) can be *expected* (i.e. consistent with the applicable product information) or *unexpected* (i.e. not consistent with such information).

An adverse event includes:

- An exacerbation of a pre-existing illness
- An increase in frequency or intensity of a pre-existing episodic event or condition
- A condition detected or diagnosed after trial medication administration even though it may have been present prior to the start of the trial
- Continuous persistent disease/symptoms present at baseline that worsen following the start of the trial

An adverse event does **NOT** include

- Medical or surgical procedures (e.g. surgery, endoscopy, tooth extraction, transfusion); the condition that leads to the procedure is an AE
- Pre-existing disease or conditions present at the start of the trial that do not worsen
- Situations where an untoward medical occurrence has not occurred (e.g. hospitalisations for cosmetic elective surgery/social/convenience admissions)
- Infection-associated conditions or signs/symptoms associated with such conditions unless more severe than expected for the subject’s condition (see also section 6.1.3)
- Overdose of either antibacterial agents or concomitant medication without any signs or symptoms
- A laboratory abnormality which is judged by the investigator not to be of clinical significance

### Definition of a Serious Adverse Event

A serious adverse event (SAE) is any event that:

- is fatal
- is life threatening (except if clearly related the patients baseline condition or medical complications to this).
- The subject was, in the view of the investigator, at immediate risk of death as the event occurred. This definition does **not** include an event that, had it occurred in a more serious form, may have caused death
- is disabling or incapacitating
- is a congenital anomaly in the offspring of a subject who received a drug (except if clearly related to the patients baseline condition or medical complications to this.
- is an event which, though not included in points above or below, may jeopardise the subject or may require intervention to prevent one of the outcomes listed in the points above (except if clearly related to the patients baseline condition or medical complications to this). Medical and scientific judgement should be used in deciding whether prompt reporting is appropriate in this situation
- requires in-patient hospitalisation or prolongs a current hospitalisation

Regarding hospitalisation:

- Hospitalisation for elective treatment of a pre-existing condition that did not worsen during the trial is **not** considered an AE
- Complications that occur during hospitalisation are AEs. If a complication prolongs hospitalisation, the event is a SAE
- “In-patient” hospitalisation means the subject has been formally admitted to a hospital for medical reasons. This may or may not be overnight. It does not include presentation at a casualty or emergency room

Note that grading of adverse events by severity (see appendix 2 and 3) – by it self – cannot be used to define a serious adverse event. A serious adverse event may be grade 3 whereas a grade 4 adverse event may not *necessarily* be a serious adverse event (although it often will be). Clinical judgement is essential to define a serious adverse event.

### Protocol-specific Exemptions from the AE and SAE Definition

The following events are not to be considered AEs or SAEs:

- any condition which occurred prior to the subject being enrolled in the trial and which did NOT worsen during the trial

## Clinical Laboratory Abnormalities and Other Abnormal Assessments as AEs and SAEs

Abnormal laboratory findings (e.g., clinical chemistry, haematology, urinalysis) or other abnormal assessments (e.g., ECGs, X-rays, vital signs) that are judged by the investigator as **clinically significant** must be recorded as AEs or SAEs if they meet the definition of an AE (see section 6.1)

- Clinically significant abnormal laboratory findings or other abnormal assessments that are detected after trial drug administration or that are present at baseline and worsen following the start of the trial are included as AEs or SAEs. However, *clinically significant abnormal laboratory findings or other abnormal assessments that are associated with the* *patients baseline condition or medical complications to this, unless judged by the investigator as more severe than expected for the subject’s condition, are* ***not*** *included as AEs or SAEs.*

The investigator should exercise his or her medical and scientific judgement in deciding whether an abnormal laboratory finding or other abnormal assessment is clinically significant.

## Recording of the AEs and SAEs in the CRF

Separate forms exist for AEs and SAEs.

For each SAE to be recorded, the following information is collected: date of onset, date of resolution, short description of event, concomitant medication, test results, the maximum grade, and the relationship with one or more of the antibacterial agent (if specific agent(s) is suspected, this is indicated).

If any of this information changes at a later time point, *a new and up-dated SAE-report must be submitted.*

## Documenting AEs and SAEs

Any AE occurring during the trial must be documented in the subject’s medical records, in accordance with the investigator’s normal clinical practice, and on the AE page of the CRF. SAEs which occur during the trial must be documented in the subject’s medical record and on the SAE page of the CRF.

A separate set of SAE pages should be used for each SAE. However, if at the time of initial reporting, multiple SAEs are present that are temporally and/or clinically related, they may be reported on the same SAE page.

The investigator should attempt to establish a diagnosis of the event based on signs, symptoms, and/or other clinical information. In such cases, *the diagnosis should be documented as the AE and/or SAE and not the individual signs/symptoms*.

If a clinically significant abnormal laboratory finding or other abnormal assessment meets the definition of an AE or SAE, then the AE CRF page or SAE CRF page must be completed, as appropriate. A diagnosis, if known, or clinical signs and symptoms if diagnosis is unknown, rather than the clinically significant abnormal laboratory finding, should be completed on AE or SAE CRF page. If no diagnosis is known and clinical signs and symptoms are not present, then the abnormal finding should be recorded. The laboratory data should either be recorded on the SAE form with the reference range and baseline value(s) or copies of the laboratory reports and reference ranges should be sent with the SAE CRF pages.

The SAE pages of the CRF should be completed as thoroughly as possible and signed by a physician before transmittal to The PASS coordinating centre. It is **very important** that the investigator provide his/her assessment of causality to any possible drug(s) at the time of the initial SAE report.

## Follow-up of AEs and SAEs

All AEs and SAEs must be followed until resolution, until the condition stabilises, until the event is otherwise explained, or the subject is lost to follow-up. The investigator is responsible to ensure that follow-up includes any supplemental investigations that may be indicated to completely evaluate the nature and/or causality of the AE or SAE. This may include additional laboratory tests or investigations, histo-pathological examinations, or consultation with other health care professionals.

The PASS coordinating centre may request that the investigator perform or arrange for the conduct of supplemental measurements and/or evaluations. If a subject dies during participation in the trial, the PASS coordinating centre should be provided with a copy of any post-mortem findings, including histo-pathology.

## Time-lines for reporting of SAEs

All adverse events that are *both serious* (see section 6.1.2 for definition) and *unexpected* (see section 6.1.1 for definition) are subject to expedited reporting by the investigator to the PASS coordinating centre. Timelines for reporting:

- *If, in addition to be serious and unexpected, the SAE is also fatal or life threatening, the following timelines for reporting should be followed:* Preliminary reporting should be made as soon as possible but no later than 15 calendar days after first knowledge by the investigator that a case qualifies, followed by as complete a report as possible within 8 additional calendar days.
- *For all other serious and unexpected SAEs, the following time line exist:* Reporting should be made as soon as possible but no later than 30 calendar days after first knowledge by the investigator that a case qualifies.
- All SAE should be reported to the PASS coordinating centre. Time lines hereof should follow those indicated above, i.e. reporting should be made as soon as possible but no later than 15 calendar days after first knowledge by the investigator that a case qualifies, followed by as complete a report as possible within 8 additional calendar days.

Facsimile transmission of the SAE CRF should always be used.

# TRIAL ADMINISTRATION

## Data Collection

Case Report Forms (CRF) will be provided for each subject by the PASS coordinating centre. All data on the CRFs must be entered legibly in black ink or typed, in Danish or English. Amendments and errors on the CRFs should not be erased, covered with correction fluid or completely crossed-out; rather, a single line should be drawn through the error and the correction initialled and dated by the investigator, authorised colleague or co-worker. An explanatory note for the change should also be written on the CRF. Any requested information which is not obtained or unanswerable should be identified by entering ‘ND’ (not done). An explanation must be documented for any missing data. CRFs must be completed regularly and should never bear the participant’s name. Participants will be identified by initials, date of birth and subject trial number only.

The investigator (or a person appointed by the investigator) must sign and date a declaration on the CRF attesting to his/her responsibility for the quality of all data recorded and that the data represents a complete and accurate record of each subject’s participation in the trial.

Details and procedures for the completion of the CRFs are specified in the Manual of Operational Procedures.

All trial CRFs will be plain paper copies – the original being the investigators copy. After completion of each page of the CRF, the investigator will send it by fax to the PASS coordinating centre. Pages will be reviewed and clarified in accordance with the protocol specific Review and Validation Manual. The data will be double entered (punched and verified) by separate data entry specialists to produce data files.

Identical validation checks will be performed on each database. Data failing any check will be flagged for output on a Data Clarification Report (DCR) and sent to the relevant investigator for resolution. In such cases the investigator is requested to sign and date any explanation or correction. On return, the database will be updated appropriately and the original DCR stored with the original CRF.

The database(s) will be subject to agreed Quality Control (QC) checks before authorisation. The data will be subsequently analysed according to the methods outlined in Section 5.

## Regulatory and Ethical Considerations

### Regulatory Authority Approval

The co-ordinator (in collaboration with the PASS coordinating centre) will obtain approval from the appropriate regulatory agency prior to initiating the trial at a site.

This trial will be conducted in accordance with ICH-GCP and all applicable regulations, including, where applicable, the Declaration of Helsinki, June 1964, as modified by 52nd WMA General Assembly, Edinburgh, Scotland, October 2000 (see Appendix 1).

### Ethics Approval

It is the investigator’s responsibility to ensure that this protocol is reviewed and approved by the appropriate local Independent Ethics Committee (IEC). The IEC must also review and approve the site’s informed consent form (ICF) and any other written information provided to the subject prior to any enrolment of subjects, and any advertisement that will be used for subject recruitment. The co-ordinator and/or the investigator must forward to the PASS coordinating centre copies of the IEC approval and the approved informed consent materials, which must be received by the PASS coordinating centre prior to the start of the trial.

If, during the trial, it is necessary to amend either the protocol or the informed consent form, the co-ordinator and/or investigator will be responsible for ensuring the IEC reviews and approves these amended documents. IEC approval of the amended ICF must be obtained before new subjects consent to take part in the trial using this version of the form. Copies of the IEC approval of the amended ICF and the approved amended ICF must be forwarded to the PASS coordinating centre as soon as available.

### Subject Informed Consent

The investigator or his/her designee will inform the subject of all aspects pertaining to the subject’s participation in the trial.

The process for obtaining subject informed consent will be in accordance with all applicable regulatory requirements. The investigator or his/her designee and the subject/ witness of an oral informed consent/ subjects legally acceptable representative must both sign and date the ICF before the subject can participate in the trial. Following types of informed consent can be accepted because of the nature of the ICU setting and the physical and/ or mental state of the subjects.

1. Ability to understand and provide written informed consent to participate in this trial,

**or**

1. Ability to understand and provide oral informed consent in presence of at least one impartial witness who should sign and personally date the consent form

**or**

1. The subjects legally acceptable representative can understand and provide written informed consent if the subject is not capable of this because of the present mental or physical condition of the subject.

The subject will receive a copy of the signed and dated form and the original will be retained in the site trial records. The decision regarding subject participation in the trial, that is made by the subject, is entirely voluntary. The investigator or his/her designee must emphasize to the subject that consent regarding trial participation may be withdrawn at any time without penalty or loss of benefits to which the subject is otherwise entitled.

If the ICF is amended during the trial, the investigator must follow all applicable regulatory requirements pertaining to approval of the amended ICF by the IEC and use of the amended form (including for ongoing subjects).

## Trial Monitoring

In accordance with applicable regulations, good clinical practice (GCP), monitors will periodically contact the site, including conducting on-site visits. The extent, nature and frequency of on-site visits will be based on enrolment rate, the quality of the documents provided by the site, consistency of follow-up of the patients according to this protocol.

During these contacts, the monitor will:

- check and assess the progress of the trial
- review trial data collected
- conduct Source Document Verification
- identify any issues and address their resolution

This will be done in order to verify that the:

- data are authentic, accurate, and complete
- safety and rights of subjects are being protected
- trial is conducted in accordance with the currently approved protocol (and any amendments), GCP, and all applicable regulatory requirements

The investigator agrees to allow the monitor direct access to all relevant documents and to allocate his/her time and the time of his/her staff to the monitor to discuss findings and any relevant issues.

In addition to contacts during the trial, the monitor will also contact the site prior to the start of the trial to discuss the protocol and data collection procedures with site personnel.

At trial closure, monitors will also conduct all activities as indicated in Section 7.5, Trial and Site Closure.

## Quality Assurance

At its discretion, the PASS coordinating centre may conduct a quality assurance audit of this trial. If such an audit occurs, the investigator agrees to allow the auditor direct access to all relevant documents and to allocate his/her time and the time of his/her staff to the auditor to discuss findings and any relevant issues. A guideline for audit is available at the PASS coordinating centre.

In addition, regulatory agencies may conduct a regulatory inspection of this trial. If such an inspection occurs, the investigator agrees to allow the inspector direct access to all relevant documents and to allocate his/her time and the time of his/her staff to the inspector to discuss findings and any relevant issues.

## Trial and Site Closure

Upon completion of the trial, the following activities, when applicable, must be conducted by the monitor in conjunction with the investigator, as appropriate:

- return of all trial data to the PASS coordinating centre
- data clarifications and/or resolutions
- review of site trial records for completeness
- shipment of stored samples to assay laboratory

In addition, the steering committee reserves the right to temporarily suspend or prematurely discontinue this trial either at a single site or at all sites at any time and for any reason. If such action is taken, selected members of the PASS steering committee and/or the PASS coordinating centre will discuss this with the Investigator (including the reasons for taking such action) at that time. The PASS coordinating centre will promptly inform all other investigators conducting the trial if the trial is suspended or terminated for safety reasons. The investigators will inform their local/regional/national regulatory authorities (as appropriate) of the suspension or termination of the trial and the reason(s) for the action. If required by applicable regulations, the investigator must inform the IEC promptly and provide the reason for the suspension or termination.

If the trial is prematurely discontinued, all trial data must be returned to the PASS coordinating centre.

## Records Retention

In accordance with applicable regulatory requirements, following closure of the trial, the investigator will maintain a copy of all site trial records in a safe and secure location. The PASS coordinating centre will inform the investigator of the time period for retaining these records in order to comply with applicable regulatory requirements.

## Information Disclosure and Inventions

### Confidentiality

The investigator and other trial site personnel will keep confidential any information provided by the co-ordinating centre (including this protocol) related to this trial and all data and records generated in the course of conducting the trial, and will not use the information, data, or records for any purpose other than conducting the trial. These restrictions do not apply to: (1) information which becomes publicly available through no fault of the investigator or trial site personnel; (2) information which it is necessary to disclose in confidence to an IEC solely for the evaluation of the trial; or (3) information which it is necessary to disclose in order to provide appropriate medical care to a trial subject.

### Publication

The findings from this trial is intended to be published in peer-reviewed journals. The steering committee decides whether abstracts are to be submitted to conferences, and how the results are distributed if more than one manuscript is to be drafted.

**Authorship**: The trial group as a whole will appear in an appendix in all published manuscripts. Co-authors are selected after a fair evaluation of primarily number of patients entered in to the trial and the level of involvement in the drafting of the manuscript. Providing that several manuscripts are to be drafted, a fair rotation among the participating clinical sites of co-authorship slots will be done taking in to consideration the number of patients enrolled.

## Indemnification and Compensation for Injury

The insurance that covers liability in relation to patient care in Denmark, *Patientforsikringen* will cover all liability aspects of the conduct of this trial^45-46^.

# REFERENCES

1: Garnacho-Montero J, Garcia-Garmendia JL, Barrero-Almodovar A, Jimenez-Jimenez FJ, Perez-Paredes C, Ortiz-Leyba C. Impact of adequate empirical antibiotic therapy on the outcome of patients admitted to the intensive care unit with sepsis. Crit Care Med 2003;3:2742-51.

2: Alberti C, Brun-Buisson C, Burchardi H, Martin C, Goodman S, Artigas A, Sicignano A, Palazzo M, Moreno R, Boulme R, Lepage E, Le Gall R. Epidemiology of sepsis and infection in ICU patients from an international

multicentre cohort study. Intensive Care Med. 2002 Feb;28(2):108-21. Epub 2001 Dec 04.

3: Alberti C, Brun-Buisson C, Goodman SV, et al. European Sepsis Group. Influence of systemic inflammatory response syndrome and sepsis on outcome of critically ill infected patients. Am J Respir Crit Care Med 2003;168:77-84. Epub 2003 Apr 17.

4. Azoulay E, Alberti C, Legendre I, Brun Buisson C, Le Gall JR. Post-ICU mortality in critically ill infected patients: an international study. Intensive Care Med. 2004 Nov 4; [Epub ahead of print]

5. Iapichino G, Morabito A, Mistraletti G, Ferla L, Radrizzani D, Reis Miranda D. Determinants of post-intensive care mortality in high-level treated critically ill patients. Intensive Care Med. 2003 Oct;29(10):1751-6. Epub 2003 Aug 16.

6. Moreno R, Miranda DR, Matos R, Fevereiro T. Mortality after discharge from intensive care: the impact of organ system failure and nursing workload use at discharge. Intensive Care Med. 2001 Jun;27(6):999-1004.

7. Azoulay E, Adrie C, De Lassence A, Pochard F, Moreau D, Thiery G, Cheval C, Moine P, Garrouste-Orgeas M, Alberti C, Cohen Y, Timsit JF. Determinants of postintensive care unit mortality: a prospective multicenter study.

Crit Care Med. 2003 Feb;31(2):428-32.

8: Assicot M, Gendrel D, Carsin H, Raymond J, Guilbaud J, Bohuon C. High serum procalcitonin concentrations in patients with sepsis and infection. Lancet 1993;341:515-8.

9. Ittner L, Born W, Rau B, Steinbach G, Fischer JA. Circulating procalcitonin and cleavage products in septicaemia compared with medullary thyroid carcinoma. Eur J Endocrinol. 2002 Dec;147(6):727-31.

10. Redl H, Schlag G, Togel E, Assicot M, Bohuon C. Procalcitonin release patterns in a baboon model of trauma and sepsis: relationship to cytokines and neopterin. Crit Care Med. 2000 Nov;28(11):3659-63.

11. Nijsten MW, Olinga P, The TH, de Vries EG, Koops HS, Groothuis GM, Limburg PC, ten Duis HJ, Moshage H, Hoekstra HJ, Bijzet J, Zwaveling JH. Procalcitonin behaves as a fast responding acute phase protein in vivo and in

vitro. Crit Care Med. 2000 Feb;28(2):458-61.

12. Chirouze C, Schuhmacher H, Rabaud C, Gil H, Khayat N, Estavoyer JM, May T, Hoen B. Low serum procalcitonin level accurately predicts the absence of bacteremia in adult patients with acute fever. Clin Infect Dis. 2002 Jul 15;35(2):156-61. Epub 2002 Jun 17. 13. Reny JL, Vuagnat A, Ract C, Benoit MO, Safar M, Fagon JY.

Diagnosis and follow-up of infections in intensive care patients: value of C-reactive protein compared with other clinical and biological variables. Crit Care Med. 2002 Mar;30(3):529-35.

14. BalcI C, Sungurtekin H, Gurses E, Sungurtekin U, Kaptanoglu B. Usefulness of procalcitonin for diagnosis of sepsis in the intensive care unit. Crit Care. 2003 Feb;7(1):85-90. Epub 2002 Oct 30.

15. Hatherill M, Tibby SM, Turner C, Ratnavel N, Murdoch IA. Procalcitonin and cytokine levels: relationship to organ failure and mortality in pediatric septic shock. Crit Care Med. 2000 Jul;28(7):2591-4.

16. Hatherill M, Tibby SM, Sykes K, Turner C, Murdoch IA. Diagnostic markers of infection: comparison of procalcitonin with C reactive protein and leucocyte count. Arch Dis Child. 1999 Nov;81(5):417-21.

17: Meisner M, Rauschmayer C, Schmidt J, et al. Early increase of procalcitonin after cardiovascular surgery in patients with postoperative complications. Intensive Care Med 2002;28:1094-102. Epub 2002 Jul 06.

18: Adamik B, Kubler-Kielb J, Golebiowska B, Gamian A, Kubler A. Effect of sepsis and cardiac surgery with cardiopulmonary bypass on plasma level of nitric oxide metabolites, neopterin, and procalcitonin: correlation

with mortality and postoperative complications. Intensive Care Med 2000;26:1259-67.

19: Lindberg M, Hole A, Johnsen H, et al. Reference intervals for procalcitonin and C-reactive protein after major abdominal surgery. Scand J Clin Lab Invest 2002;62:189-94.

21: Aouifi A, Piriou V, Blanc P, et al. Effect of cardiopulmonary bypass on serum procalcitonin and C-reactive protein concentrations. Br J Anaesth. 1999;83:602-7.

22: Conlon JM, Grimelius L, Thim L. Structural characterization of a high-molecular-mass form of calcitonin

[procalcitonin-(60-116)-peptide] and its corresponding N-terminal flanking peptide [procalcitonin-(1-57)-peptide] in a human medullary thyroid carcinoma. Biochem J 1988;256:245-50.

23: Birnbaum RS, Mahoney WC, Burns DM, O'Neil JA, Miller RE, Roos BA. Identification of procalcitonin in a rat medullary thyroid carcinoma cell line. J Biol Chem 1984;259:2870-4.

24: Jacobs JW, Lund PK, Potts JT Jr, Bell NH, Habener JF. Procalcitonin is a glycoprotein. J Biol Chem 1981;256:2803-7.

25: Becker KL, Nylen ES, White JC, Muller B, Snider RH Jr. Clinical review 167: Procalcitonin and the calcitonin gene family of peptides in inflammation, infection, and sepsis: a journey from calcitonin back to its precursors. J Clin Endocrinol Metab. 2004 Apr;89(4):1512-25. Review. No abstract available.

26: Linscheid P, Seboek D, Nylen ES, Langer I, Schlatter M, Becker KL, Keller U, Muller B. In vitro and in vivo calcitonin I gene expression in parenchymal cells: a novel product of human adipose tissue. Endocrinology. 2003 Dec;144(12):5578-84. Epub 2003 Aug 21.

27: Linscheid P, Seboek D, Schaer DJ, Zulewski H, Keller U, Muller B. Expression and secretion of procalcitonin and calcitonin gene-related peptide by adherent monocytes and by macrophage-activated adipocytes. Crit Care Med. 2004 Aug;32(8):1715-21.

28: Meisner M, Muller V, Khakpour Z, Toegel E, Redl H. Induction of procalcitonin and proinflammatory cytokines in an anhepatic baboon endotoxin shock model. Shock 2003;19:187-90.

29: Dandona P, Nix D, Wilson MF, et al. Procalcitonin increase after endotoxin injection in normal subjects.

J Clin Endocrinol Metab 1994;79:1605-8.

30: Christ-Crain M, Jaccard-Stolz D, Bingisser R, Gencay MM, Huber PR, Tamm M, Muller B. Effect of procalcitonin-guided treatment on antibiotic use and outcome in lower respiratory tract infections: cluster-randomised, single-blinded intervention trial. Lancet. 2004 Feb 21;363(9409):600-7.

31: Jensen J, Heslet L, Jensen TH, Espersen K, Steffensen P, Tvede M. Procalcitonin increase identifies critically ill patients at high risk of mortality. Submitted 26. January 2005.

32: Vesentini S, Bassi C, Talamini G, Cavallini G, Campedelli A, Pederzoli P. Prospective comparison of C-reactive protein level, Ranson score and contrast-enhanced computed tomography in the prediction of septic complications of acute pancreatitis. Br J Surg 1993;80:755-7.

33: Reny JL, Vuagnat A, Ract C, Benoit MO, Safar M, Fagon JY. Diagnosis and follow-up of infections in intensive care patients: value of C-reactive protein compared with other clinical and biological variables. Crit Care Med 2002;30:529-35.

34. Assay Characteristics, BRAHMS diagnostica, Hennigsdorf, Germany.

35: Meisner M, Lohs T, Huettemann E, Schmidt J, Hueller M, Reinhart K. The plasma elimination rate and urinary secretion of procalcitonin in patients with normal and impaired renal function. Eur J Anaesthesiol. 2001 feb;18(2):79-87.

36 Fleischhack G, Kambeck I, Cipic D, Hasan C, Bode U. Procalcitonin in paediatric cancer patients: its diagnostic relevance is superior to that of C-reactive protein, interleukin 6, interleukin 8, soluble interleukin 2 receptor and soluble tumour necrosis factor receptor II. Br J Haematol. 2000 Dec;111(4):1093-102.

37: von Lilienfeld-Toal M, Dietrich MP, Glasmacher A, Lehmann L, Breig P, Hahn C, Schmidt-Wolf IG, Marklein G, Schroeder S, Stuber F. Markers of bacteremia in febrile neutropenic patients with haematological malignancies: procalcitonin and IL-6 are more reliable than C-reactive protein. Eur J Clin Microbiol Infect Dis. 2004 Jul;23(7):

539-44. Epub 2004 Jun 22.

38: Giamarellos-Bourboulis EJ, Grecka P, Poulakou G, Anargyrou K, Katsilambros N, Giamarellou H. Assessment of procalcitonin as a diagnostic marker of underlying infection in patients with febrile neutropenia. Clin Infect Dis. 2001 Jun 15;32(12):1718-25. Epub 2001 May 21.

39: Persson L, Engervall P, Magnuson A, Vikerfors T, Soderquist B, Hansson LO, Tidefelt U. Use of inflammatory markers for early detection of bacteraemia in patients with febrile neutropenia. Scand J Infect Dis. 2004;36(5):365-71.

40: Giamarellou H, Giamarellos-Bourboulis EJ, Repoussis P, Galani L, Anagnostopoulos N, Grecka P, Lubos D, Aoun M, Athanassiou K, Bouza E, Devigili E, Krcmery V, Menichetti F, Panaretou E, Papageorgiou E, Plachouras D. Potential use of procalcitonin as a diagnostic criterion in febrile neutropenia: experience from a multicentre study.

Clin Microbiol Infect. 2004 Jul;10(7):628-33.

41: Barnes C, Ignjatovic V, Newall F, Carlin J, Ng F, Hamilton S, Ashley D, Waters K, Monagle P. Change in serum procalcitonin (deltaPCT) predicts the clinical outcome of children admitted with febrile neutropenia. Br J Haematol. 2002 Sep;118(4):1197-8. No abstract available.

42: Odamaki M, Kato A, Kumagai H, Hishida A. Counter-regulatory effects of procalcitonin and indoxyl sulphate on net albumin secretion by cultured rat hepatocytes. Nephrol Dial Transplant. 2004 Apr;19(4):797-804.

43: Nakae H, Inaba H, Endo S. Usefulness of procalcitonin in Pseudomonas burn wound sepsis model. Tohoku J Exp Med. 1999 Jul;188(3):271-3.

44: Holzheimer RG. Oral antibiotic prophylaxis can influence the inflammatory response in aortic aneurysm repair: results of a randomized clinical study. J Chemother. 2003 Apr;15(2):157-64.

45. Danish Law regulation 1997-03-24 nr. 228 about patient insurance

46. www.patientforsikringen.dk

.

Table 1: Clinical and laboratory Evaluations

| Evaluation | Day  (screening & baseline) | | Day (counting after admission to ICU)  (follow-up) | | | | |
| --- | --- | --- | --- | --- | --- | --- | --- |
|  | 1 | Day=Dis-charge/ death | 28 | 30 | 60 | 90 | 180 |
| Informed Consent | X |  |  |  |  |  |  |
| Entry Criteria | X |  |  |  |  |  |  |
| Demography | X |  |  |  |  |  |  |
| APACHE II | X | X |  |  |  |  |  |
| Infections during this hospital admission | X |  |  |  |  |  |  |
| Current medical conditions | X | X |  |  |  |  |  |
| State of daily function and health | X |  |  | X |  |  | X |
| Mortality |  | (X) | X |  | X | X | X |
| Baseline PCT | X |  |  |  |  |  |  |
| AUC_procalcitonin_ |  | X |  |  |  |  |  |
| Concurrent Medications^a^ | X | X |  | X | X | X | X |
| Haematology | X | X |  |  |  |  |  |
| Clinical chemistry | X | X |  |  |  |  |  |
| Adverse events | X^a^ | X |  |  |  |  |  |
| Serious Adverse Events | X^a^ | X |  | X | X | X | X |

a Adverse events and serious adverse events are registered daily

1. APPENDICES

**Appendix 1**

Declaration of Helsinki

**WORLD MEDICAL ASSOCIATION DECLARATION OF HELSINKI**

**Ethical Principles for Medical Research Involving Human Subjects**

Adopted by the 18th WMA General Assembly
Helsinki, Finland, June 1964
and amended by the
29th WMA General Assembly, Tokyo, Japan, October 1975
35th WMA General Assembly, Venice, Italy, October 1983
41st WMA General Assembly, Hong Kong, September 1989
48th WMA General Assembly, Somerset West, Republic of South Africa, October 1996
and the
52nd WMA General Assembly, Edinburgh, Scotland, October 2000

1. **INTRODUCTION**
2. The World Medical Association has developed the Declaration of Helsinki as a statement of ethical principles to provide guidance to physicians and other participants in medical research involving human subjects. Medical research involving human subjects includes research on identifiable human material or identifiable data.
3. It is the duty of the physician to promote and safeguard the health of the people. The physician's knowledge and conscience are dedicated to the fulfillment of this duty.
4. The Declaration of Geneva of the World Medical Association binds the physician with the words, "The health of my patient will be my first consideration," and the International Code of Medical Ethics declares that, "A physician shall act only in the patient's interest when providing medical care which might have the effect of weakening the physical and mental condition of the patient."
5. Medical progress is based on research which ultimately must rest in part on experimentation involving human subjects.
6. In medical research on human subjects, considerations related to the well-being of the human subject should take precedence over the interests of science and society.
7. The primary purpose of medical research involving human subjects is to improve prophylactic, diagnostic and therapeutic procedures and the understanding of the aetiology and pathogenesis of disease. Even the best proven prophylactic, diagnostic, and therapeutic methods must continuously be challenged through research for their effectiveness, efficiency, accessibility and quality.
8. In current medical practice and in medical research, most prophylactic, diagnostic and therapeutic procedures involve risks and burdens.
9. Medical research is subject to ethical standards that promote respect for all human beings and protect their health and rights. Some research populations are vulnerable and need special protection. The particular needs of the economically and medically disadvantaged must be recognized. Special attention is also required for those who cannot give or refuse consent for themselves, for those who may be subject to giving consent under duress, for those who will not benefit personally from the research and for those for whom the research is combined with care.
10. Research Investigators should be aware of the ethical, legal and regulatory requirements for research on human subjects in their own countries as well as applicable international requirements. No national ethical, legal or regulatory requirement should be allowed to reduce or eliminate any of the protections for human subjects set forth in this Declaration.
11. **BASIC PRINCIPLES FOR ALL MEDICAL RESEARCH**
12. It is the duty of the physician in medical research to protect the life, health, privacy, and dignity of the human subject.
13. Medical research involving human subjects must conform to generally accepted scientific principles, be based on a thorough knowledge of the scientific literature, other relevant sources of information, and on adequate laboratory and, where appropriate, animal experimentation.
14. Appropriate caution must be exercised in the conduct of research which may affect the environment, and the welfare of animals used for research must be respected.
15. The design and performance of each experimental procedure involving human subjects should be clearly formulated in an experimental protocol. This protocol should be submitted for consideration, comment, guidance, and where appropriate, approval to a specially appointed ethical review committee, which must be independent of the investigator, the sponsor or any other kind of undue influence. This independent committee should be in conformity with the laws and regulations of the country in which the research experiment is performed. The committee has the right to monitor ongoing trials. The researcher has the obligation to provide monitoring information to the committee, especially any serious adverse events. The researcher should also submit to the committee, for review, information regarding funding, sponsors, institutional affiliations, other potential conflicts of interest and incentives for subjects.
16. The research protocol should always contain a statement of the ethical considerations involved and should indicate that there is compliance with the principles enunciated in this Declaration.
17. Medical research involving human subjects should be conducted only by scientifically qualified persons and under the supervision of a clinically competent medical person. The responsibility for the human subject must always rest with a medically qualified person and never rest on the subject of the research, even though the subject has given consent.
18. Every medical research project involving human subjects should be preceded by careful assessment of predictable risks and burdens in comparison with foreseeable benefits to the subject or to others. This does not preclude the participation of healthy volunteers in medical research. The design of all studies should be publicly available.
19. Physicians should abstain from engaging in research projects involving human subjects unless they are confident that the risks involved have been adequately assessed and can be satisfactorily managed. Physicians should cease any investigation if the risks are found to outweigh the potential benefits or if there is conclusive proof of positive and beneficial results.
20. Medical research involving human subjects should only be conducted if the importance of the objective outweighs the inherent risks and burdens to the subject. This is especially important when the human subjects are healthy volunteers.
21. Medical research is only justified if there is a reasonable likelihood that the populations in which the research is carried out stand to benefit from the results of the research.
22. The subjects must be volunteers and informed participants in the research project.
23. The right of research subjects to safeguard their integrity must always be respected. Every precaution should be taken to respect the privacy of the subject, the confidentiality of the patient's information and to minimize the impact of the study on the subject's physical and mental integrity and on the personality of the subject.
24. In any research on human beings, each potential subject must be adequately informed of the aims, methods, sources of funding, any possible conflicts of interest, institutional affiliations of the researcher, the anticipated benefits and potential risks of the study and the discomfort it may entail. The subject should be informed of the right to abstain from participation in the study or to withdraw consent to participate at any time without reprisal. After ensuring that the subject has understood the information, the physician should then obtain the subject's freely-given informed consent, preferably in writing. If the consent cannot be obtained in writing, the non-written consent must be formally documented and witnessed.
25. When obtaining informed consent for the research project the physician should be particularly cautious if the subject is in a dependent relationship with the physician or may consent under duress. In that case the informed consent should be obtained by a well-informed physician who is not engaged in the investigation and who is completely independent of this relationship.
26. For a research subject who is legally incompetent, physically or mentally incapable of giving consent or is a legally incompetent minor, the investigator must obtain informed consent from the legally authorized representative in accordance with applicable law. These groups should not be included in research unless the research is necessary to promote the health of the population represented and this research cannot instead be performed on legally competent persons.
27. When a subject deemed legally incompetent, such as a minor child, is able to give assent to decisions about participation in research, the investigator must obtain that assent in addition to the consent of the legally authorized representative.
28. Research on individuals from whom it is not possible to obtain consent, including proxy or advance consent, should be done only if the physical/mental condition that prevents obtaining informed consent is a necessary characteristic of the research population. The specific reasons for involving research subjects with a condition that renders them unable to give informed consent should be stated in the experimental protocol for consideration and approval of the review committee. The protocol should state that consent to remain in the research should be obtained as soon as possible from the individual or a legally authorized surrogate.
29. Both authors and publishers have ethical obligations. In publication of the results of research, the investigators are obliged to preserve the accuracy of the results. Negative as well as positive results should be published or otherwise publicly available. Sources of funding, institutional affiliations and any possible conflicts of interest should be declared in the publication. Reports of experimentation not in accordance with the principles laid down in this Declaration should not be accepted for publication.
30. **ADDITIONAL PRINCIPLES FOR MEDICAL RESEARCH COMBINED WITH MEDICAL CARE**
31. The physician may combine medical research with medical care, only to the extent that the research is justified by its potential prophylactic, diagnostic or therapeutic value. When medical research is combined with medical care, additional standards apply to protect the patients who are research subjects.
32. The benefits, risks, burdens and effectiveness of a new method should be tested against those of the best current prophylactic, diagnostic, and therapeutic methods. This does not exclude the use of placebo, or no treatment, in studies where no proven prophylactic, diagnostic or therapeutic method exists.
33. At the conclusion of the study, every patient entered into the study should be assured of access to the best proven prophylactic, diagnostic and therapeutic methods identified by the study.
34. The physician should fully inform the patient which aspects of the care are related to the research. The refusal of a patient to participate in a study must never interfere with the patient-physician relationship.
35. In the treatment of a patient, where proven prophylactic, diagnostic and therapeutic methods do not exist or have been ineffective, the physician, with informed consent from the patient, must be free to use unproven or new prophylactic, diagnostic and therapeutic measures, if in the physician's judgement it offers hope of saving life, re-establishing health or alleviating suffering. Where possible, these measures should be made the object of research, designed to evaluate their safety and efficacy. In all cases, new information should be recorded and, where appropriate, published. The other relevant guidelines of this Declaration should be followed.

# Appendix 2: Abbreviations

| **AE** | **Adverse Event (AE)** |
| --- | --- |
| **ALAT** | **Alanine Aminotransferase (SGOT)** |
| **APACHE II** | **Acute Physiology And Chronic Health Evaluation II** |
| **ASAT** | **Aspartate Aminotransferase (SGPT)** |
| **CDC** | **Centers for Disease Control** |
| **CRF** | **Case Report Form** |
| **DDD** | **Defined Day Doses** |
| **DIC** | **Disseminated Intravascular Coagulation** |
| **DSMB** | **Data Safety Monitoring Board** |
| **ICU** | **Intensive Care Unit** |
| **IEC** | **Independent Ethics Committee** |
| **IL-6** | **Interleukin 6** |
| **MODS** | **Multi Organ Dysfunction Syndrome** |
| **PASS** | **Procalcitonin and Surivival Study** |
| **PCT** | **Procalcitonin** |
| **SAE** | **Serious Adverse Event** |
| **TNFα** | **Tumor Necrosis Factor α** |
| **WBC** | **White Blood cell Count** |

# Appendix 3: Table of conversion factors for laboratory units

| **TEST** | **CONVENTIONAL** | | **SI** | |
| --- | --- | --- | --- | --- |
|  | **Unit** | **Factor** | **Unit** | **Factor** |
| Haemoglobin | g/dl | 0,6206 | mmol/l | 1,61 |
| Platelets | Thou/mm^3^ | 0,001 | ^a^x10^9^/l | 1000 |
| Hyponatraemia  (↓ Sodium)  Hypernatraemia  (↑ Sodium) | mEq/l  mEq/l | 1,0  1,0 | mmol/l  mmol/l | 1,0  1,0 |
| Hypokalaemia  (↓ Potassium)  Hyperkalaemia  (↑ Potassium) | mEq/l  mEq/l | 1,0  1,0 | mmol/l  mmol/l | 1,0  1,0 |
| Hypoglycaemia  (↓ Glucose)  Hyperglycaemia  (↑ Glucose) | mg/dl  mg/dl | 0,0555  0,0555 | mmol/l  mmol/l | 18,0  18,0 |
| Hypocalcaemia  (↓ Calcium)  Hypercalcaemia  (↑ Calcium) | mg/dl  mg/dl | 0,2495  0,2495 | mmol/l  mmol/l | 4,0  4,0 |

^a^ No SI unit

For example: Haemoglobin 9,5 g/dl - multiply by factor 0,6206 → 5,9 mmol/l

Appendix 4: Table with the used antibacterial and antifungal drugs used in the 6 participating Intensive Care Units.

| **Generic name** | **Comercial name (s)** |
| --- | --- |
| **Benzyl-Penicillin** | **Penicillin”Leo”, Penicillin”Rosco” Benzyl-Penicillin”Panpharma”** |
| **Phenoxymethyl-Penicillin** | **Calcipen ®, Pancillin ®, Primcillin ®, Rocilin ®, Vepicombin ®”DAK”** |
| **Dicloxacillin** | **Dicillin ®, Diclocil ®** |
| **Flucloxacillin** | **Heracillin** |
| **Amoxicillin** | **Amoxicillin”NM”, Flemoxin Solutab ®, Imacillin ®, Imadrax ®,** |
| **Amoxicillin+Clavulanic Acid** | **Bioclavid, Bioclavid Forte, Spektramox ®** |
| **Ampicillin** | **Ampicillin”Vepidan”, Doktacillin, Pentrexyl ®** |
| **Piperacillin** | **Ivacin ®, Pipril** |
| **Piperacillin+Tazobactam** | **Tazocin ®** |
| **Pivampicillin** | **Pondocillin ®** |
| **Pivmecillinam/ Mecillinam** | **Selexid ®** |
| **Cefalexin** | **Keflex ®** |
| **Cefalotin** | **Keflin ®** |
| **Cefepim** | **Maxipime ®** |
| **Cefotaxim** | **Claforan ®** |
| **Ceftazidim** | **Fortum ®** |
| **Ceftriaxon** | **Rocephalin ®** |
| **Cefuroxim** | **Zinacef, Cefuroxim Stragen, Zinnat ®** |
| **Aztreonam** | **Azactam ®** |
| **Meropenem** | **Meronem ®** |
| **Imipenem+cilastatin** | **Tienam ®** |
| **Azithromycin** | **Zitromax ®** |
| **Clarithromycin** | **Klacid ®, Klacid ® Uno, Klaricid, Zeclar** |
| **Erythromycin** | **Abboticin ®, Abboticin ® Novum, Erycin ®, Escumycin, Hexabotin ®** |
| **Roxithromycin** | **Surlid ®, Forimycin ®, Roximstad, Roxithromycin“Copyfarm”, Roxithromycin“UNP”** |
| **Doxycyclin** | **Vibradox ®** |
| **Lymecyclin** | **Tetralysal ®** |
| **Oxytetracyclin** | **Oxytetral ®** |
| **Tetracyclin** | **Tetracyclin“AL”, Tetracyclin“DAK”, Tetracyclin“SAD”** |
| **Gentamicin** | **Garamycin ®, Gentacoll ®, Hexamycin, Septopal, Septopal Mini** |
| **Netilmicin** | **Netilyn** |
| **Tobramycin** | **Nebcina ®, Tobi ®** |
| **Moxifloxacin** | **Avelox** |
| **Ciprofloxacin** | **Ciproxin ®, Cifin, Ciprofloxacin“1A Farma”, Ciprofloxacin“2K Pharma”, Ciprofloxacin“Alpharma”, Ciprofloxacin“Biochemie”, Ciprofloxacin“Gea”, Ciprofloxacin“Ratiopharm”, Sancipro, Sibunar ®** |
| **Ofloxacin** | **Tarivid ®** |
| **Norfloxacin** | **Zoroxin ®** |
| **Methenamin** | **Haiprex** |
| **Nitrofurantoin** | **Nitrofurantoin”DAK”, Nitrofurantoin”SAD”** |
| **Sulfamethizol** | **Lucosil ®, Sulfametizol”SAD”, Sulfametizol”Ophtha”** |
| **Trimethoprim** | **Monotrim ®, Trimethoprim”1A Farma”, Trimopan** |
| **Sulfamethoxazol+Trimethoprim** | **Sulfamethoxazol+Trimethoprim”SAD”, Sulfotrim ®** |
| **Clindamycin** | **Dalacin ®** |
| **Colistin** | **Colimycin** |
| **Teicoplanin** | **Targocid ®** |
| **Vancomycin** | **Vancocin, Vancomycin”Abbott”, Vancomycin”Alpharma”** |
| **Fusidinsyre** | **Fucidin ®** |
| **Linezolid** | **Zyvoxid ®** |
| **Metronidazol** | **Flagyl ®, Metronidazol”Alpharma”, Metronidazol”DAK”, Metronidazol”SAD”** |
| **Amphotericin B** | **Abelcet, AmBisome, Fungizone** |
| **Caspofungin** | **Cancidas ®** |
| **Fluconazol** | **Conasol, Diflucan ®, Fluconazol”Alpharma”, Fluconazol”Copyfarm”, Fluconazol”Nycomed”, Fluconazol”Ratiopharm”, Fluconazol”Stada”, Fungal ®, Fungustatin** |
| **Flucytosin** | **Ancotil** |
| **Ketoconazol** | **Nizoral ®** |
| **Voriconazol** | **Vfend** |
| **Ethambutol** | **Myambutol ®** |
| **Isoniacid** | **Isoniacid”OBA”** |
| **Pyrazinamid** | **Pyrazinamid”Medic”, Pyrazinamid”SAD”** |
| **Rifabutin** | **Rifabutin”Pharmacia”** |
| **Rifampicin** | **Rimactan ®** |
