# Supplementary material for: The Potential of Antimicrobials to Induce Thrombocytopenia in Critically Ill Patients: Data from a Randomized Controlled Trial
Source: PLoS One. 2013 Nov 28;8(11):e81477. doi: 10.1371/journal.pone.0081477 (PMC3842947; doi:10.1371/journal.pone.0081477)
Supplement: File S2 — Original statistical analysis plan. (DOCX) [file pone.0081477.s003.docx]

**File S2. Original statistical analysis plan**

**Coagulation caused Organ Malfunction: Prediction Associated with Sepsis Survival**

**(COM:PASS) – A research project that investigates the effect of antibiotics on the coagulation system including the platelets and the endothelium**

**Database study:**

The potential of antimicrobials to induce thrombocytopenia in critically ill patients: data from a randomized controlled trial

**BACKGROUND**

Thrombocytopenia in septic patients

Thrombocytopenia is the most common coagulation disorder in critically ill patients and a major concern in the intensive care unit (ICU) (Rice et al. 2009). Thrombocytopenia is associated with prolonged hospitalization and reduced survival rates (Vandijck et al. 2010). In addition, a study including 56 ICU patients with sepsis showed that thrombocytopenia is bad, but not recovering from thrombocytopenia is worse (**Faviere et al. 2011).**

Thrombocytopenia in ICU patients is most often caused by a severe life-threatening bacterial infection (severe sepsis and septic shock). Up to 56% of septic patients admitted to the Intensive Care Unit (ICU) have been reported to have thrombocytopenia (Strauss et al. 2002). In these patients, thrombocytopenia is usually thought to be caused by the condition (consumption, sequestering, destruction and decreased production due to bone marrow depression). In addition the drugs given to treat the condition, including various antimicrobial drugs, can cause thrombocytopenia (destruction and dilution). Several antibiotics have been reported to cause thrombocytopenia among then Beta-lactamase , Vancomycin, Fluorquinolones and Fungicides (Amfotericin B, azols) (Al-Nouri et al. 2012).

However the effect of different antibiotics on the platelet count has, to our knowledge, never been investigated in a randomized controlled trial.

**Definitions**

ICU: Intensive Care Unit

High-ex: High exposure to antibiotic (the intervention group)

SOC: Standard-of-care

Day 1:Baseline, defined as admission to the ICU

**Overall hypothesis:**

Antibiotics affect the platelet count in critically ill patients causing thrombocytopenia

**Aim**

1. Investigate factors associated with developing thrombocytopenia over day 1-28.

We hypothesize such factors as 1) severe sepsis/septic shock at baseline, 2) randomization group, 3) APACHE II at baseline, 4) age, 5) type of antibiotic received while in the ICU, 6) gender, 7) surgical patient “yes” or “no” 8) platelet count at baseline 9) BMI 10) Charlson score

1. Investigate whether thrombocytopenia anytime (absolute and/or dynamic) predicts 28-day mortality.

**METHODS**

**Data Source**

The Procalcitonin And Survival Study (PASS) ([1](#_ENREF_1)) is a multicentre, randomized controlled trial in Denmark 2006 to 2009 in critically ill patients. 1200 patients were randomized (1:1) either to a pro-active antibiotic strategy guided by biomarker levels (high-exposure group) vs. a standard-of-care antibiotic strategy (SOC group) ([2](#_ENREF_2), [3](#_ENREF_3)). Daily information regarding microbiology, biochemistry, radiology, physiology and clinical assessment were registered. In addition type and daily prescribed dosage of antimicrobial therapy was collected.

Patients were followed until death or day 28 and the primary result from the study was a comparable mortality rate between the two randomized groups (overall 28-day mortality was 38,1%).

Infection status at randomization was similar in the two groups and 37% (433 patients) had severe sepsis or septic shock.

Previous analysis showed that the patients in the high-exposure group received significantly more piperacillin/tazobactam and ciprofloxacin compared with the SOC group. Vancomycin was used to a lesser extent in both groups. The median length of an antibiotic course was prolonged using the high-exposure algorithm (6 days vs. 4 days).

After the primary analyses, investigators realized that the data collected would be useful to investigate how the platelet count is affected when critically ill patients are exposed to a large amount of broad-spectrum antibiotic. The study design made it possible to compare the platelets counts in the two arms as the platelet count and the infection status at randomization were similar between the two randomized groups.

**Analyses**

**Aim 1.**

a)

Quadratic mixed model with random intercept and slope

Mixed effect model with response platelet count in the raw scale over day 1-28 with the quadratic term for time. Covariates to be considered:

1. Treatment arm (high exposure vs. SOC)
2. age (≥65 vs. <65 years)
3. gender (male vs. female)
4. baseline APACHE II score (≥20 vs <20)
5. Severe sepsis/septic shock at baseline
6. Surgical patients “yes” or “no”
7. current exposure to a specific antibiotic* (time-dependant)
8. BMI
9. Charlson score

Interaction between the above covariates and time is investigated.

For covariates showing an interaction with time, the estimate for the intercept, linear and quadratic term of time (per day) will be tabulated by group, both from the unadjusted model and from the model adjusted for all other covariates. For the covariate treatment arm, randomization holds and therefore no further adjustment is required.

*Antibiotics are: piperacillin /tazobactam, cefuroxime, ciprofloxacin and meropenem.

A latent class mixed model joint with time-to-event data will maybe be considered.

b)

Survival analysis

In all patients evaluate time-to-thrombocytopenia defined as first time there is a 20% drop in platelet count from baseline or from maximum value ever previously achieved.

In the subset of those without thrombocytopenia at baseline evaluate time-to-thrombocytopenia defined as platelet count below an absolute level of 100 x 10^9^ (or alternatively 150 x 10^9^). In this analysis patients whose last measurement was before day 7 and 28 will be censored at that point in an alternative analysis. We will define as event those who dropped out because of death. Kaplan-Meier curves will be used to estimate cumulative risk of achieving each of the endpoints stratified by covariates 1-9 above (except platelet count at baseline). Multivariable analysis will be conducted using Cox regression analysis. Univariable analysis will be performed for the treatment arm only.

**Aim2.**

Survival analysis – time-to-death

3 analysis are performed using the following exposure factors; baseline platelet count, current platelet count, current change in platelet count (dynamic). Time will accrue from randomization date to time of death or to day 28. We assume that treatment is not modified by current platelet count and therefore there is no time-dependant confounding affected by prior treatment and results from the Cox model are interpretable. The Cox models will be adjusted for the covariates 1-10 mentioned above.

Maria Egede Johansen , On behalf of the project group
